# Longitudinal plasma proteomics predict phenoconversion to clinically manifest ALS

**DOI:** 10.1038/s41591-026-04528-x

**Published:** 2026-07-27

**Authors:** Ximing Ran, Joanne Wuu, Zhaohui S. Qin, Michael P. McDermott, Johnathan Cooper-Knock, Yindi Li, Volkan Granit, Anne-Laure Grignon, Elizabeth Lin, Maria Catalina Fernandez, Danielle Colato, Nathan Carberry, Christina M. Lill, Paolo Piazza, Andrea Malaspina, Michael Benatar

**Affiliations:** 1 Department of Biostatistics and Bioinformatics, Emory University, Atlanta, GA USA; 2 Department of Computer Science, Emory University, Atlanta, GA USA; 3 Department of Neurology and ALS Center, University of Miami Miller School of Medicine, Miami, FL USA; 4 Department of Biostatistics and Computational Biology, University of Rochester School of Medicine and Dentistry, Rochester, NY USA; 5 Department of Neurology, University of Rochester School of Medicine and Dentistry, Rochester, NY USA; 6 Sheffield Institute for Translational Neuroscience (SITraN), University of Sheffield, Sheffield, UK; 7 NIHR Sheffield Biomedical Research Centre, Sheffield, UK; 8 Department of Neuromuscular Diseases, UCL Queen Square Motor Neuron Disease Center, UCL Queen Square Institute of Neurology, University College London, London, UK; 9 Institute of Epidemiology and Social Medicine, University of Münster, Münster, Germany; 10 Ageing and Epidemiology Unit (AGE), School of Public Health, Imperial College London, London, UK; 11 Nuffield Department of Medicine, Centre for Human Genetics, University of Oxford, Oxford, UK

**Keywords:** Biomarkers, Amyotrophic lateral sclerosis

## Abstract

The study of pre-symptomatic amyotrophic lateral sclerosis (ALS) and the design of disease prevention trials are greatly hampered by our inability to predict which unaffected carriers of ALS-associated pathogenic variants will phenoconvert to clinically manifest disease and when. In this longitudinal Olink Explore, high-throughput, proteomic study, 516 serially collected plasma samples from 33 phenoconverters, 35 patients with ALS, 10 pre-symptomatic pathogenic variant carriers and 59 controls were included. Here we identified 92 proteins with concentrations that changed before phenoconversion; characterized the longitudinal trajectory of these proteins and identified a core panel of 19 proteins which, collectively, predicted phenoconversion over the 0.5-year to 5-year time horizons (crossvalidated areas under the curve 0.80–0.89) and yielded estimates of time to phenoconversion with a mean absolute error of 1.6 years. These findings were partially replicated in UK Biobank data, confirming pre-symptomatic increases in several proteins (for example, NEFL, EDA2R and CA3) and that a multi-protein panel outperformed NEFL alone in estimating time to phenoconversion. This work sheds light on the biology of pre-symptomatic ALS. Moreover, our identification of a panel of new susceptibility/risk biomarkers based on empirical longitudinal data furthers the ultimate goal of ALS prevention.

## Abstract (web summary)

Longitudinal proteomic analysis of plasma from unaffected carriers of ALS-associated pathogenic variants revealed early protein changes before ALS symptoms and enabled mapping of their trajectories as well as prediction of the timing of phenoconversion

## Main

Historically, neurodegenerative diseases such as amyotrophic lateral sclerosis (ALS), frontotemporal dementia (FTD), Parkinson’s disease and Alzheimer’s disease have been defined based on the presence of a characteristic clinical phenotype. Increasingly, however, these disorders are being defined based on their underlying biology^1–3^, with the recognition that there are both pre-symptomatic and clinically manifest stages of disease. Indeed, there is a growing recognition that early, even pre-symptomatic, intervention is likely to offer the greatest prospect for meaningful therapeutic effect. As a result, a seismic shift—from a traditional care paradigm to prevention—is underway^4–10^.

Although prevention of the onset of biologically defined disease might be an aspirational goal, in ALS and FTD emerging efforts are currently focused on preventing phenoconversion to clinical manifestations^7^. A major obstacle to such prevention trials is our limited ability to predict when phenoconversion is likely to occur and in whom^8^. The identification of a pre-symptomatic increase in blood neurofilament light chain (NfL) concentration^11,12^ was essential for the design and implementation of ATLAS, the first ALS prevention trial^13^. In the context of this trial, NfL serves as a susceptibility or risk biomarker, predicting risk of phenoconversion at the individual level^14,15^. Importantly, the observed rise in NfL in the 6–12 months before phenoconversion was apparent in carriers of highly penetrant *SOD1* pathogenic variants associated with rapidly progressive disease^11^. NfL, however, reflects the rate of axonal loss^16^ and thus NfL alone may be insufficient for the prediction of phenoconversion among carriers of a broader array of pathogenic variants, especially those that are more slowly progressive^12,17^. Also, as NfL reflects axonal loss, a consequence of upstream pathobiological mechanisms of disease, biomarkers reflecting these mechanisms are to be expected. The discovery and validation of additional susceptibility/risk biomarkers are, therefore, critical to the feasibility and success of future disease prevention trials.

Biofluid-based biomarkers in ALS and FTD hold great promise for a host of reasons. First, unlike imaging or physiologically based biomarkers, which largely capture downstream manifestations of disease, biochemical biomarkers have the potential to elucidate the underlying pathobiology of disease. Second, as biofluid-based biomarkers reflect systemic, as opposed to regional or topographically restricted, manifestations of disease, they offer a window into early pathobiological changes irrespective of the anatomical location of disease. Third, there is broad agreement on, and widespread implementation of, standard operating procedures for the collection, processing and storage of biological samples for biomarker development. Finally, emerging omics platforms that have been subjected to rigorous analytical validation permit large-scale and relatively unbiased discovery efforts. Crucial to the endeavor of developing susceptibility/risk biomarkers is the availability of a cohort of individuals (that is, phenoconverters) who have been prospectively followed over an extended period of time—from the pre-symptomatic state through phenoconversion to clinically manifest disease—and in whom the timing of symptom onset is known and clearly defined. Practically, carriers of pathogenic variants associated with substantially elevated risk for ALS and/or FTD are currently the only population in whom such natural history studies are feasible.

Over the course of the past ~18 years, the Pre-Symptomatic Familial ALS (Pre-fALS) study^18^ has followed a large cohort of unaffected pathogenic variant carriers at substantially elevated risk for ALS and/or FTD (hereinafter ALS/FTD), with longitudinal data and sample acquisition both before and after phenoconversion to clinically manifest disease. Combined with data and biospecimens similarly collected from controls and those with clinically manifest ALS in companion studies, the Pre-fALS cohort offers an unprecedented opportunity for the discovery of susceptibility/risk biomarkers to aid the prediction of phenoconversion. Here we report findings from the application of the Olink Explore HT platform to plasma samples from this unique cohort, with independent replication using data from the UK Biobank^19^.

## Results

### Discovery cohort

This proteomic study included participants from three parent studies: Pre-fALS, the Clinical Research in ALS (CRiALS) Biomarker study and the CReATe Consortium Phenotype–Genotype–Biomarker (PGB1) study. Details of Pre-fALS have previously been described^11,12,18,20,21^. Briefly, Pre-fALS is a single-center study that recruits, from across North America, individuals who are carriers of any ALS (and ALS/FTD)-associated pathogenic variant and who, at the time of enrollment, are clinically pre-symptomatic. CRiALS Biomarker is a companion study to Pre-fALS, serving to recruit both healthy controls and patients with clinically manifest ALS, to aid the interpretation of pre-symptomatic data from Pre-fALS. CRiALS Biomarker study procedures and clinical assessments mirror those used in Pre-fALS. Additional participants with clinically manifest ALS were drawn from CReATe PGB1, a multi-center natural history study of individuals with ALS and related disorders, in which participants were evaluated serially to acquire longitudinal phenotypic data and biospecimens. PGB1 participants included in this report were all from the University of Miami site. (See Methods for additional details.)

### Participant characteristics

**Extended Data Fig. 1:**
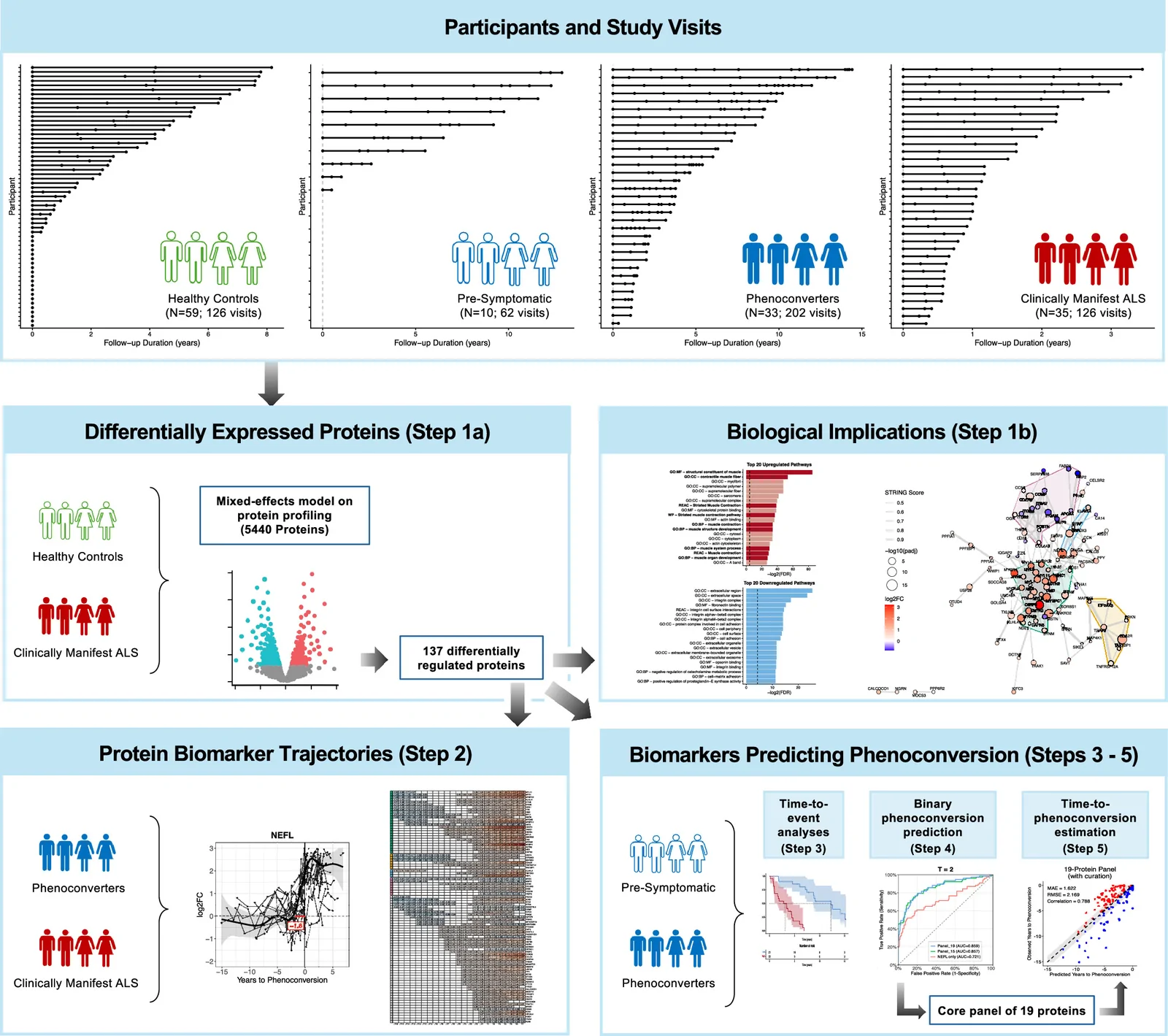
Discovery cohort study schema. Using longitudinal data from controls, pre-symptomatic carriers, phenoconverters and those with clinically manifest ALS, the analyses proceeded through five steps: (1) We identified 137 proteins that are differentially regulated between ALS and controls, and summarized their biological implications. (2) Using combined data from phenoconverters and those with clinically manifest ALS, we characterized the longitudinal trajectory of protein biomarkers. (3-5) Using data from pre-symptomatic carriers and phenoconverters, we performed time-to-event analyses, phenoconversion event prediction and phenoconversion timing estimation to identify susceptibility/risk biomarkers that predict phenoconversion.

For the discovery cohort, 516 plasma samples from *n* = 137 participants were included. The phenoconverter group comprised *n* = 33 Pre-fALS participants who had phenoconverted to clinically manifest ALS and/or FTD, and from whom plasma samples and accompanying phenotypic data were available before (*n* = 33) and after (*n* = 23) phenoconversion (total 202 visits). To provide context and facilitate interpretation of data, we also included samples and data from *n* = 59 controls (126 visits), *n* = 35 patients with ALS (126 visits) and *n* = 10 pre-symptomatic pathogenic variant carriers who had not yet phenoconverted (62 visits) (Extended Data Fig. 1). With the exception of *n* = 21 controls, all participants contributed longitudinal samples from multiple visits. Demographic and clinical characteristics are summarized in Table 1.

**Table 1:** Demographic and clinical characteristics

Characteristic		Discovery cohort (University of Miami)				Replication cohort (UK Biobank)				
		Healthy control	Pre-symptomatic	Phenoconverter	Clinically manifest ALS	Healthy control	Pre-symptomatic	Phenoconverter^a^	Pre-hospital^a^	Clinically manifest ALS
Number of participants	*n*	59	10	33	35	35,722	38	231	33	22
Sex, male	*n* (%)	27 (46)	6 (60)	13 (39)	20 (57)	16,733 (47)	15 (40)	123 (53)	18 (55)	15 (68)
Baseline age, years	Mean (±s.d.)	51.3 (±12.4)	49.1 (±8.9)	57.2 (±10.1)	56.8 (±9.4)	56.7 (±8.2)	55.1 (±6.3)	60.8 (±6.8)	61.4 (±6.5)	60.6 (±6.5)
Genotype group										
*SOD1* A4V	*n* (%)	–	4 (40)	15 (46)	1 (3)	0	0	0	0	0
*SOD1* non-A4V	*n* (%)	–	4 (40)	8 (24)	2 (6)	0	4 (11)	1 (0.4)	1 (3)	0
*C9orf72*	*n* (%)	–	2 (20)	7 (21)	6 (17)	0	34 (89)	24 (11)	8 (24)	2 (9)
Other genes	*n* (%)	–	–	3 (9)^b^	0	0	–	3 (1)^c^	0	0
None identified	*n* (%)	–	–	0	26 (74)	35,722	–	203 (88)	24 (73)	20 (91)
Years from onset to baseline (that is, first sampling)	Median (range)	–	–	–	1.55 (0–4.85)	–	–	–	–	3.61 (0.09–17.23)
Number of participants with longitudinal visits	*n*	38	10	33	35	0	0	0	0	0
Total number of visits	*n*	126	62	140^d^	126	35,722	38	231	33	22
Number of visits per person^e^	Median (range)	3 (2–4)	6.5 (2–10)	5 (2–15)	3 (2–7)	–	–	–	–	–
Follow-up duration, years	Median (range)	3.39^e,f^ (0.29–8.16)	10.71^g^ (3.53–15.24)	4.36^h^ (0.13–15.07)	1.05^f^ (0.33–3.45)	16.46^g^ (0.11–19.47)	16.36^g^ (2.43–18.13)	4.13^h^ (0.05–11.88)	1.35^i^ (0.15–1.98)	–

–, Not applicable

^a^For UK Biobank, phenoconversion was estimated to be 2 years before the first record of hospitalization with an ICD-10 of G12.2. Participants with baseline sampling before and after this estimated phenoconversion date were included in the phenoconversion and pre-hospitalization groups, respectively.

^b^Including one *FUS*, one*TARDBP* and one *VCP*.

^c^Including one *ANXA11*, one *OPTN* and one *VCP*.

^d^In addition to 140 pre-phenoconversion visits, *n* = 23 phenoconverters also contributed 62 post-phenoconversion visits.

^e^Among those with longitudinal visits.

^f^Time from baseline to last sampling.

^g^Time from baseline to censoring (that is, data cutoff).

^h^Time from baseline to phenoconversion (discovery cohort) or proxy phenoconversion (replication cohort).

^i^Time from baseline to first record of hospitalization with an ICD-10 of G12.2.

### Disease state biomarkers and implicated biological pathways

**Fig. 1:**
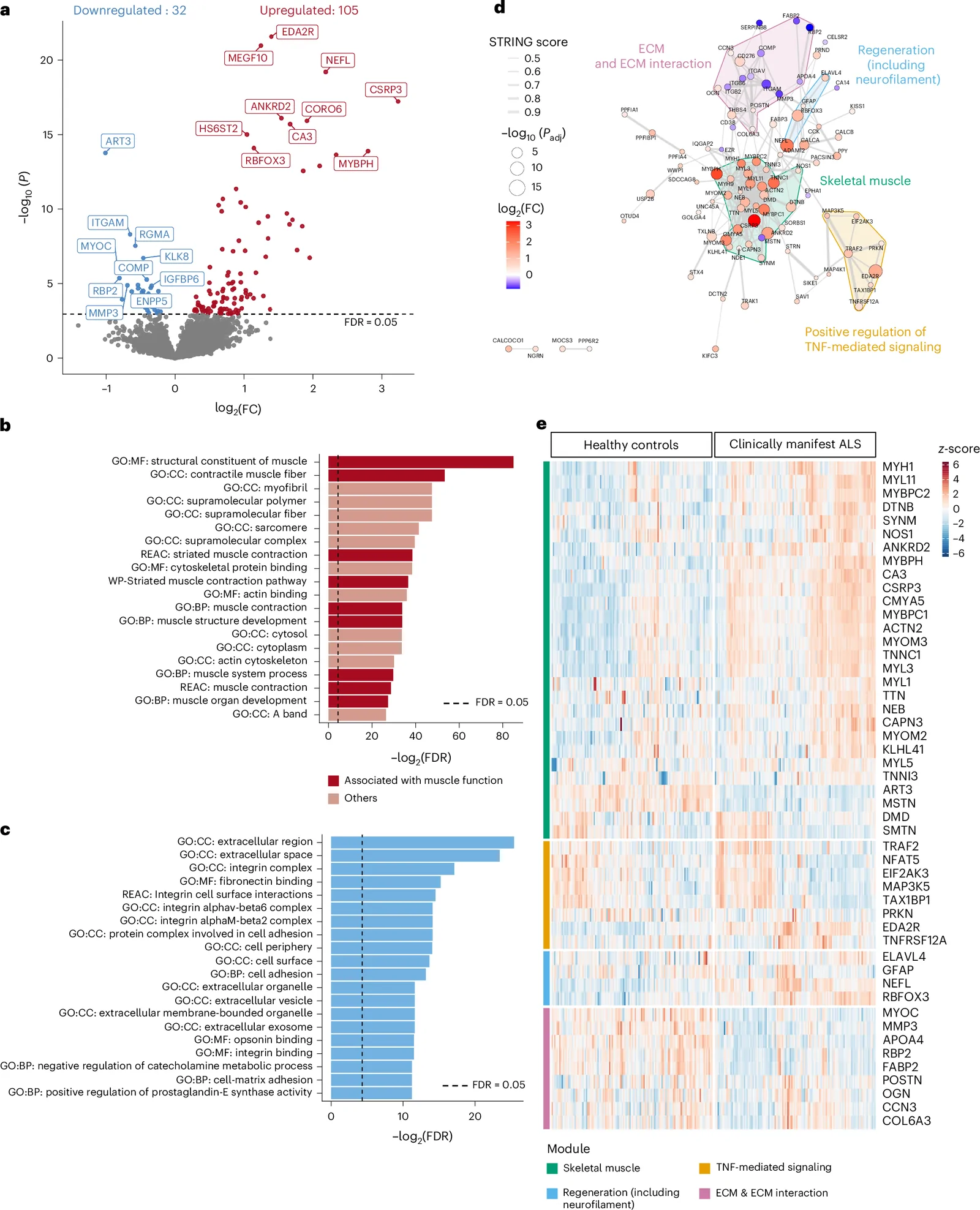
Disease state biomarkers. **a**, Volcano plot illustrating differentially regulated proteins between clinically manifest ALS and healthy controls. Significantly upregulated proteins are shown in red and significantly downregulated proteins in blue. The log_2_(fold-change), or log_2_(FC), represents the estimated fixed-effect coefficient (for ALS versus controls) from the linear mixed-effects model. *P* values, obtained from same model, were based on two-sided *t*-tests and adjusted using the Benjamini–Hochberg FDR procedure. The dashed black line indicates the FDR threshold of 0.05. **b**, The top 20 pathways that are most enriched among upregulated proteins. Dark-red shading and bold font highlight pathways associated with muscle function. The dashed black line indicates the FDR threshold of 0.05. GO, gene ontology; MF, molecular function; CC, cellular component; BP, biological process; REAC, Reactome Pathway Database; WP, WikiPathways. **c**, The top 20 pathways that are most enriched among downregulated proteins. The dashed black line indicates the FDR threshold of 0.05. **d**, Protein–protein interaction network of differentially regulated proteins, with clusters related to skeletal muscle (green), the ECM (purple), regeneration and neurofilament (blue), and positive regulation of TNF-mediated signaling (gold) highlighted. The log_2_(FC) represents the estimated fixed-effect coefficient (for ALS versus controls) from the linear mixed-effects model. Adjusted *P* values (*P*_adj_), obtained from same model, were based on two-sided *t*-tests and adjusted using the Benjamini–Hochberg FDR procedure. **e**, Heatmap of 49 proteins with distinct expression patterns, of which 44 corresponded to proteins highlighted in **d**. Five additional proteins (ART3, CA3, SMTN, NFAT5 and MYOC) that lacked protein–protein interaction edges in **d** are also included here. The columns represent data from individual study participants and the rows indicate specific proteins.

The Olink Explore HT panel includes 5,440 proteins. After multiple quality control (QC) steps, data from 5,298 proteins were retained. Applying a mixed-effects model to compare clinically manifest ALS with healthy controls, we identified 137 differentially expressed proteins, of which 105 were upregulated and 32 downregulated (false discovery rate (FDR) = 0.05) (Fig. 1a and Supplementary Table 1). Gene ontology (GO) term^22^ enrichment analysis identified skeletal muscle as the dominant pathway implicated by upregulated proteins (Fig. 1b) and extracellular matrix (ECM) as the dominant pathway implicated by downregulated proteins (Fig. 1c). Protein–protein interaction network analysis^23^ identified clusters related to skeletal muscle function, the ECM, positive regulation of tumor necrosis factor (TNF)-mediated signaling and regeneration (which includes neurofilament) (Fig. 1d). A heatmap depiction of expression patterns of a subset of 49 (out of the 137) differentially regulated proteins illustrates the distinct molecular signatures associated with disease status (Fig. 1e). Parenthetically, in this report we use the nonitalicized gene name to refer to the protein product (for example, NfL is denoted by NEFL, the gene that encodes it). We adopted this convention for ease of visualization and to enable comparability to the published literature.

### Temporal course of biomarkers across pre-symptomatic and clinically manifest ALS

**Fig. 2:**
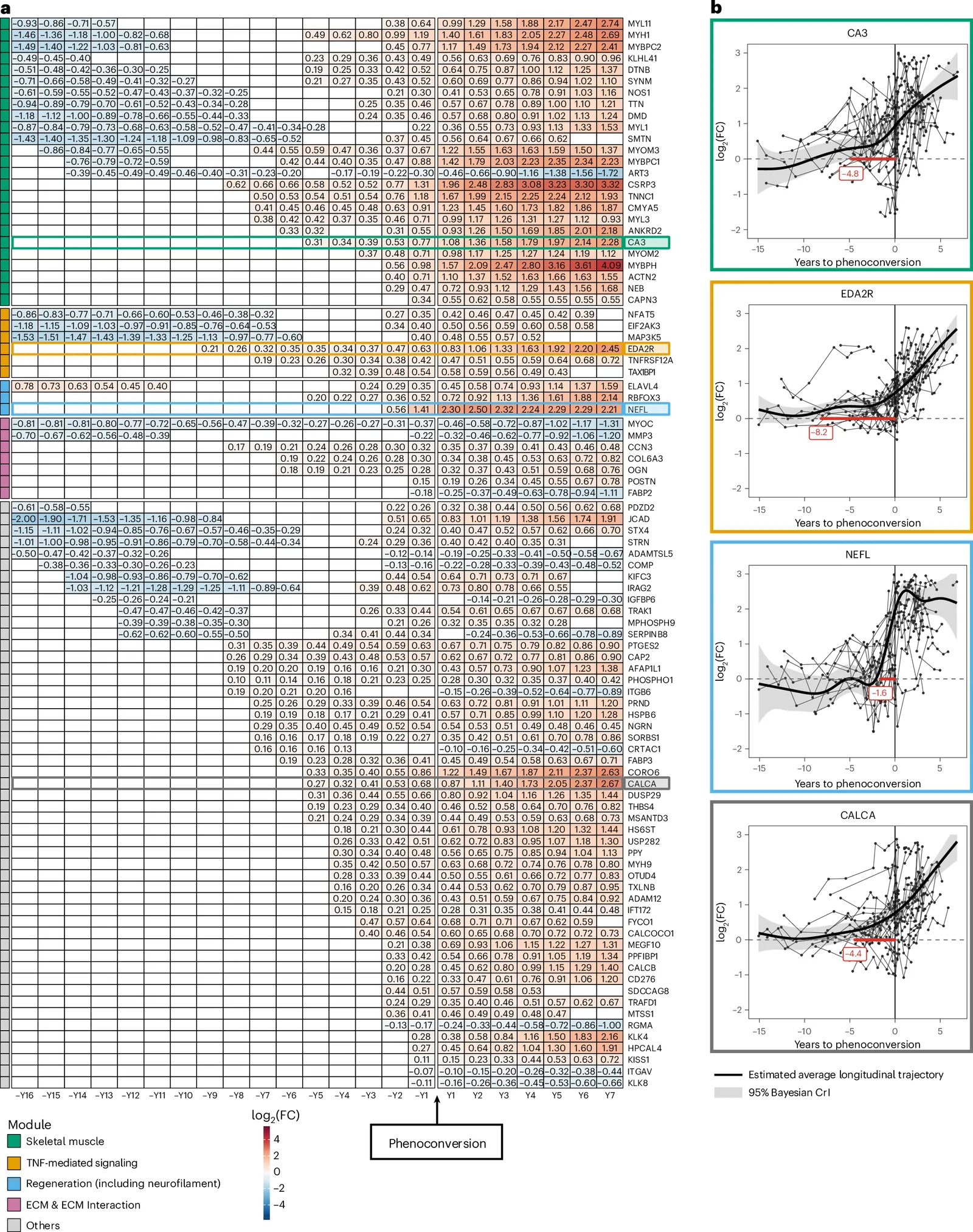
Longitudinal trajectory of protein biomarkers before and after phenoconversion. **a**, Heatmap depicting 92 proteins with significant changes in their relative abundance, compared to age- and sex-matched controls, during the pre-symptomatic stage of disease. The color scale reflects the direction and magnitude of change, with warmer colors (red) indicating increased concentrations and cooler colors (blue) decreased concentration relative to controls. The number in each cell represents the average relative protein abundance during the indicated time interval before or after phenoconversion. Empty cells denote time intervals where differences, compared to controls, were not statistically significant. Proteins are grouped by functional modules (Fig. 1d), as indicated by the color bar in the first column. **b**, Longitudinal trajectories of four proteins (CA3, EDA2R, NEFL and CALCA), to further illustrate relative protein abundance compared to controls, in log_2_(FC), during the periods before and after phenoconversion. In addition to phenoconverters, participants with clinically manifest ALS were included; their estimated date of symptom onset was used as a proxy for date of phenoconversion. The dots and connecting lines show the longitudinal data from individual participants. The black curve depicts the estimated mean longitudinal trajectory of protein abundance, compared to age- and sex-matched controls in the first layer of generalized additive mixed model (GAMM); the shaded band denotes the 95% Bayesian credible interval (CrI) around the fitted mean trend. Horizontal red lines indicate the time periods during which relative protein abundance is significantly elevated (that is, log_2_(FC) > 0).

**Extended Data Fig. 2:**
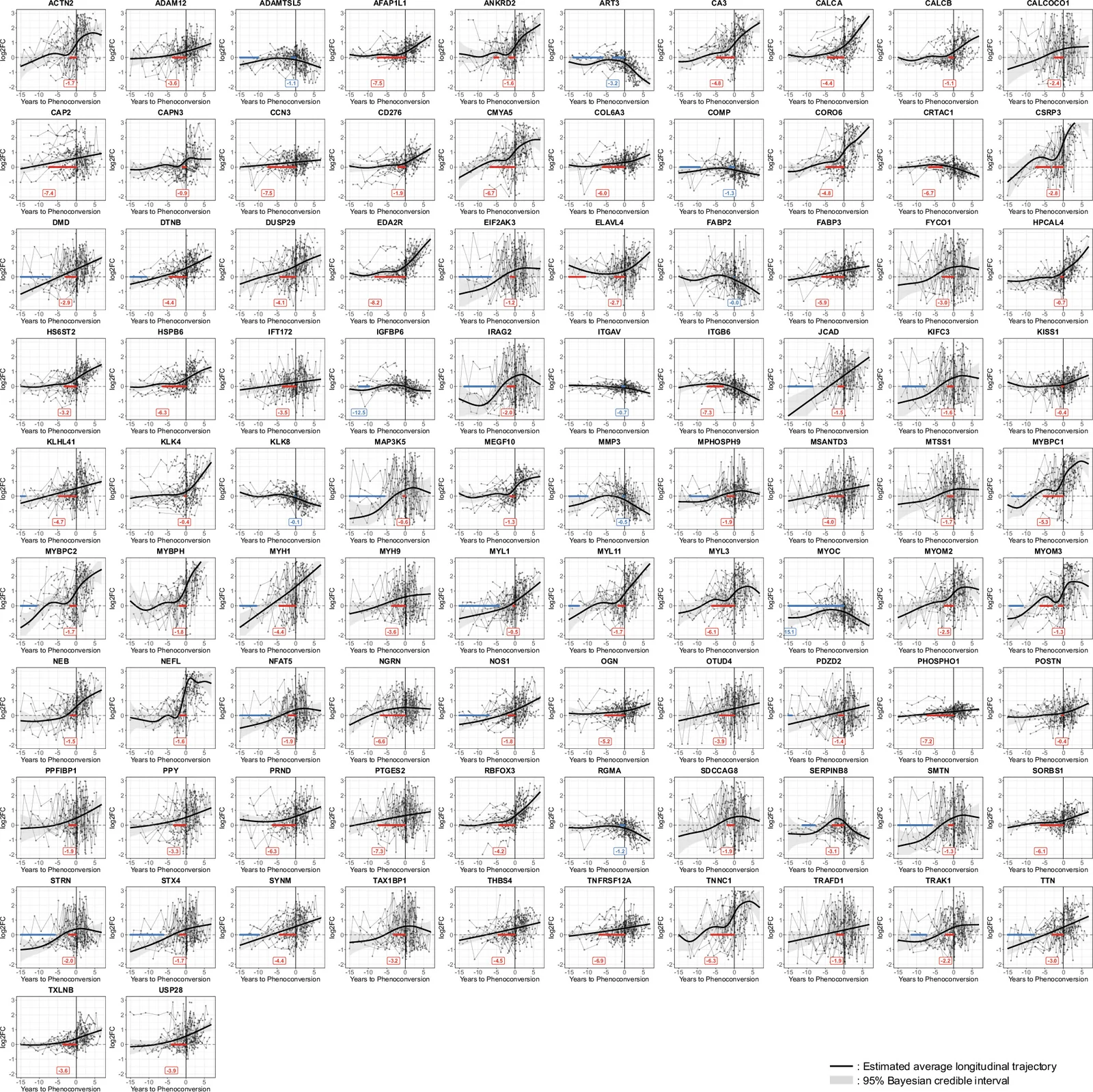
Protein biomarker trajectories: phenoconverters and clinically manifest ALS. Trajectories of 92 protein biomarkers that changed pre-symptomatically, based on data from phenoconverters (pre- and post-conversion) and clinically manifest ALS. For the clinically manifest ALS group, estimated date of symptom onset was used as a proxy for date of phenoconversion. Trajectories were derived from generalized additive mixed model (GAMM) analyses. The Y-axis denotes the log2FC in protein relative to age- and sex-matched healthy controls by first layer GAMM. The dots and connecting lines show the longitudinal data from individual participants. The black curve depicts the estimated mean longitudinal trajectory of protein abundance; the shaded band denotes the 95% Bayesian credible interval around the fitted mean trend. Horizontal red and blue lines indicate the time periods during which protein levels of phenoconverters were, respectively, significantly higher (log2FC > 0) or lower (log2FC < 0) compared to matched controls by first layer GAMM. The numeric labels mark the approximate time at which the significant deviation first appeared.

**Extended Data Fig. 3:**
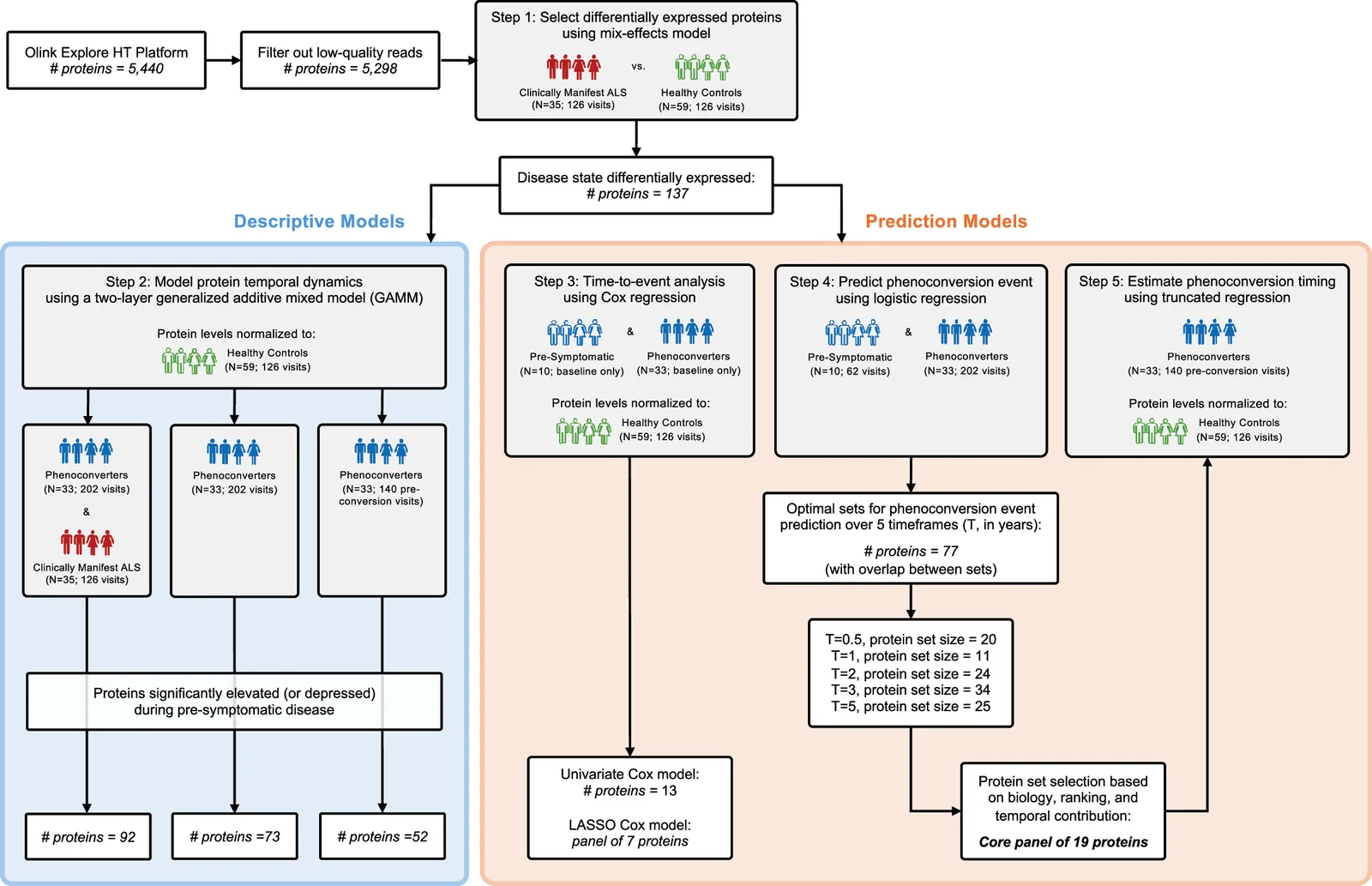
Protein selection process for discovery cohort. Flowchart summarizing the stepwise procedures for: identifying proteins that were differentially expressed based on disease state (ALS vs. controls); modeling the longitudinal trajectories of individual protein biomarkers and how they changed before and after ALS phenoconversion; modeling time-to-phenoconversion; and identifying a core panel of proteins for phenoconversion event prediction as well as phenoconversion timing estimation.

The longitudinal trajectory of biomarkers before and after phenoconversion to clinically manifest ALS was examined using data from the phenoconverter and clinically manifest groups. Of the 137 differentially regulated proteins, 92 showed significant changes in their relative abundance (compared to age- and sex-matched controls) before phenoconversion (Fig. 2 and Extended Data Fig. 2). A similar trajectory analysis using data from phenoconverters only (including visits before and after phenoconversion) yielded 73 proteins with significant pre-symptomatic change (Supplementary Fig. 1a,b). The same analysis using only phenoconverter data from before phenoconversion yielded 52 proteins with significant change (Supplementary Fig. 1c,d). Procedures for protein selection are summarized in Extended Data Fig. 3 (left panel). Although the three approaches yielded different numbers of proteins, among proteins that showed pre-symptomatic changes in at least two approaches, the initial time at which a significant change was observed was largely comparable: across the proteins; the maximal difference in this initial time between approaches (that is, latest initial time − earliest initial time) had a median (25th–75th percentile) of 1.3 (0.7–2.1) years before phenoconversion, with a small number of outliers (8 proteins had maximal difference >10 years) (Supplementary Table 2).

Results confirmed prior observations that NEFL levels sharply rise shortly before phenoconversion and continue to increase, albeit more gradually thereafter. In addition, we observed that the concentration of numerous other proteins (for example, CA3 and EDA2R) increased many years before phenoconversion and with further increases during the clinically manifest stage of disease. For a much smaller number of proteins (for example, ART3), the concentration begins to decline before phenoconversion and with further reduction after symptom onset.

### Time-to-event analysis

**Fig. 3:**
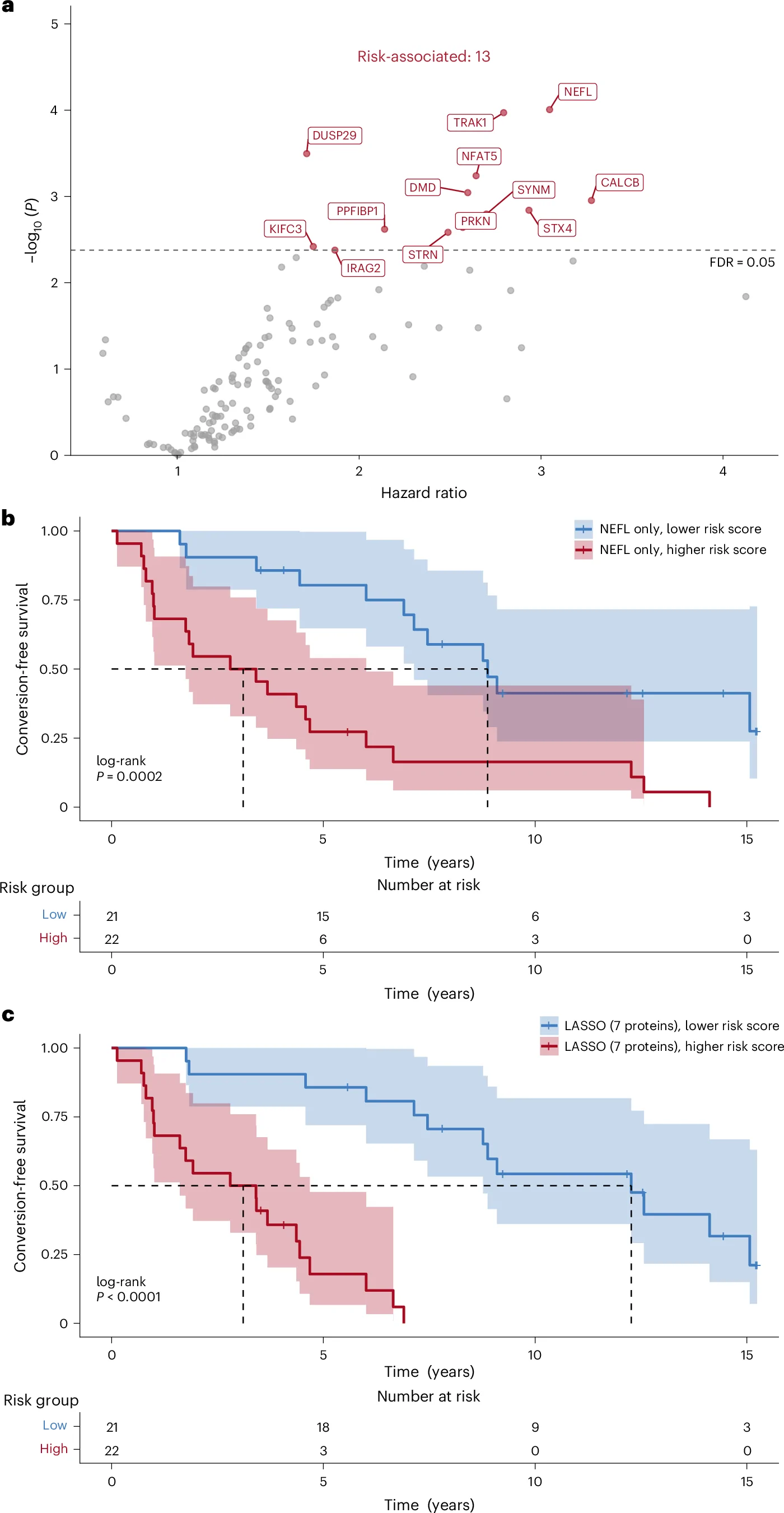
Time-to-phenoconversion models based on protein biomarkers at baseline. **a**, Volcano plot showing the association between baseline relative protein abundance, normalized to age- and sex-matched controls in a first-layer GAMM and the hazard of phenoconversion estimated from Cox proportional hazards models adjusted for age, sex and genotype. Each point represents a protein. On the *x* axis are hazard ratios per unit increase in relative protein abundance on the log_2_ scale (that is, per each doubling of protein abundance relative to controls). On the *y* axis are two-sided Wald’s *z*-test *P* values, on the −log_10_ scale, for the protein-abundance coefficient from a Cox proportional hazards model adjusted for age, sex and genotype. Proteins above the FDR threshold (<0.05) are highlighted and labeled in red. **b**,Kaplan–Meier curves showing phenoconversion-free survival stratified by baseline NEFL risk score (median split) from Cox model. Individuals in the high-risk group exhibited shorter conversion-free survival compared to those in the low-risk group (median 3.1 versus 8.9 years; log-rank *P* = 0.0002). Shaded areas represent 95% confidence intervals (CIs). **c**, Kaplan–Meier curves stratified by the baseline seven-protein, LASSO-derived risk score (median split). The high-risk group showed markedly shorter conversion-free survival compared to the low-risk group (median 3.1 versus 12.3 years; log-rank *P* = 5.32 × 10^−8^). Shaded areas represent 95% CIs.

To establish which of the 137 differentially regulated proteins between ALS and controls were associated with time to phenoconversion, we employed Cox proportional hazards models, using baseline protein biomarker value (relative protein abundance) and adjusting for clinical covariates (age, sex and genotype). Univariate models with FDR correction identified 13 proteins (all upregulated) associated with an increased hazard of phenoconversion: NEFL, DUSP29, CALCB, NFAT5, PPFIBP1, SYNM, DMD, IRAG2, STX4, KIFC3, PRKN, STRN and TRAK1 (Fig. 3a and Extended Data Table 1). Cox regression with least absolute shrinkage and selection operator (LASSO) penalty identified seven proteins significantly associated with an increased hazard of phenoconversion: NEFL, DUSP29, CALCB, NFAT5, PPFIBP1, NDE1 and CLEC4G. Kaplan–Meier analysis showed that individuals with higher (above-median) baseline NEFL risk scores had a median phenoconversion-free survival of 3.1 years compared with 8.9 years in the group with lower (below-median) NEFL risk scores (log(rank *P*) = 0.0002; Fig. 3b). The seven-protein LASSO risk score showed even stronger separation—with median survival of 3.1 years in the higher (above-median LASSO risk score) risk group compared to 12.3 years in the lower (below-median LASSO risk score) risk group (log(rank *P*) < 0.0001; Fig. 3c), indicating better predictive performance than NEFL alone. As biomarker levels may change over time, and especially as phenoconversion approaches, we also evaluated both univariate and penalized (LASSO) time-dependent Cox models. Apart from NEFL in the LASSO analysis, no protein was significantly associated with time to phenoconversion in these models, perhaps due to the limited sample size and nonuniform time intervals between visits, which constrained the application of these more complex models.

### Phenoconversion event prediction

As disease prevention trials would need to enrich for pathogenic carriers at high short-term risk of phenoconversion, we set about building models to predict whether a pre-symptomatic pathogenic variant carrier will phenoconvert to ALS within a certain timeframe. To this end, we evaluated the performance of seven machine learning-based classification methods using data from the 137 differentially regulated proteins. We restricted the analysis to these 137 proteins to limit the search space given the modest number of phenoconverters, while recognizing that this strategy primarily focused on proteins with monotonic changes over time. We included proteomic data from the pre-symptomatic group and the phenoconverter group (see Methods, step 4, for details) and considered five different time horizons—that is, whether the participant phenoconverted within 0.5, 1, 2, 3 or 5 years after any given sample collection—with annotation of visits as ‘cases’ or ‘controls’ as described in Methods. The number of ‘cases’ and ‘controls’ and their group affiliation are summarized in Extended Data Table 2. This approach leveraged the unique longitudinal structure of our data; rather than simply predicting whether an individual participant will or will not phenoconvert, this analytical approach enabled us to assess the likelihood of phenoconversion within a specified timeframe given the proteomic profile at each visit. By considering visit-level (instead of person-level) data, we were able to maximize the sample size of our training data to develop better classification models. Optimal fivefold crossvalidation areas under the curve (AUCs) derived from the seven machine learning algorithms are summarized in Supplementary Fig. 2.

**Fig. 4:**
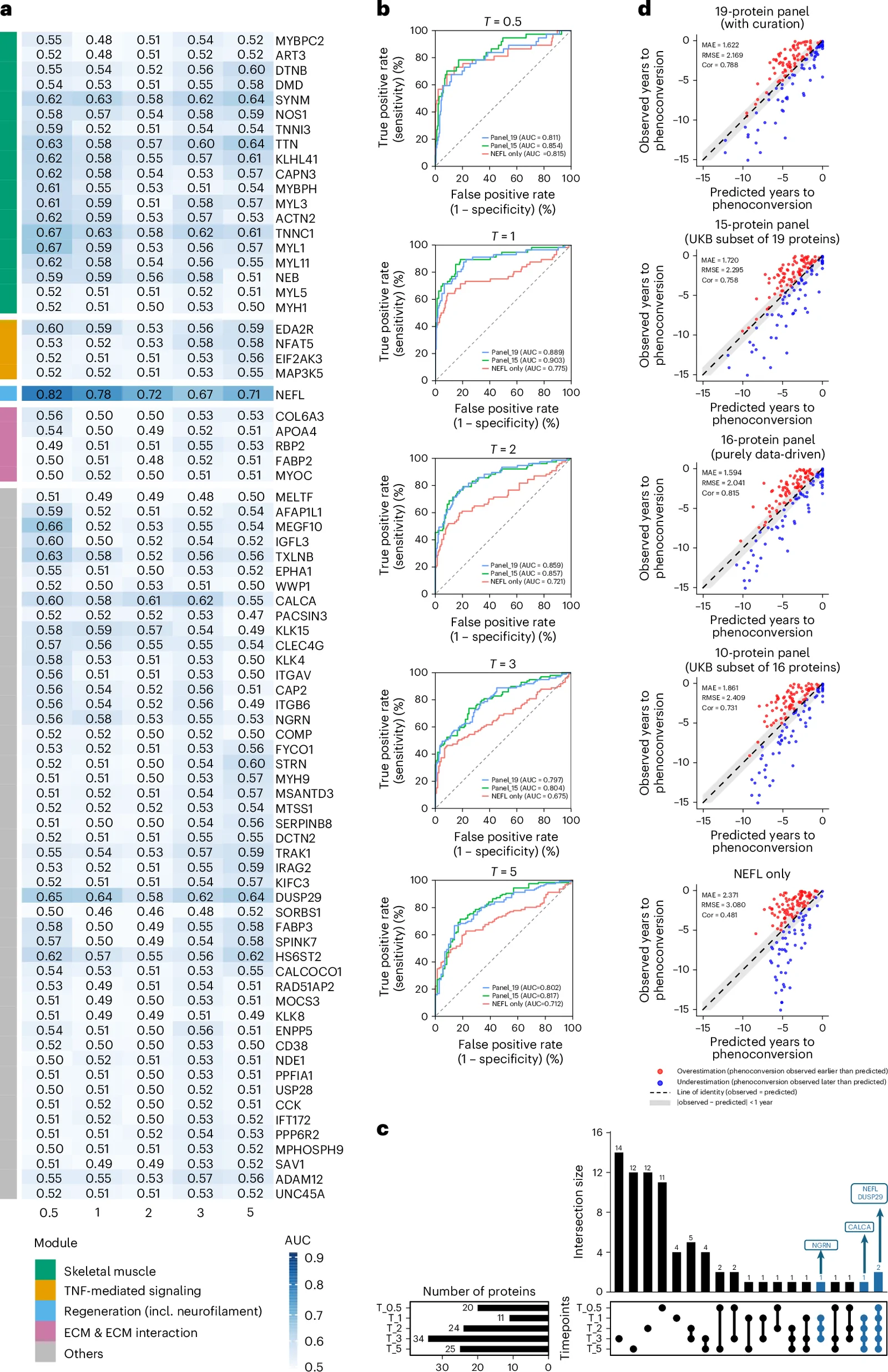
Phenoconversion event prediction and timing estimation. **a**, Heatmap illustrating the AUC of ROC curves obtained, via logistic regression, for each of the 77 proteins identified as predicting phenoconversion. Analyses were conducted across five different timeframes (0.5, 1, 2, 3 and 5 years). Each AUC value was obtained from fivefold crossvalidation. Darker shades of blue indicate higher AUCs. Proteins are grouped by functional modules (Fig. 1d), as indicated by the color bar in the first column. **b**, ROC curves comparing the performance of phenoconversion event prediction of three different models. Blue lines correspond to results obtained using the 19-protein panel (data driven with expert curation). Green lines correspond to results obtained using the 15-protein panel (subset of 19 proteins that is also available in UK Biobank data). Red lines correspond to prediction results obtained using NEFL alone. **c**, UpSet plot of protein sets identified by logistic regression to predict phenoconversion over the five timeframes. Horizontal bar chart (lower left) summarizes the number of proteins included in the optimal model for each timeframe, with NEFL and DUSP29 contributing to phenoconversion event prediction across all five timeframes. **d**, Scatterplots illustrating the relationship between observed and estimated (or predicted) time to phenoconversion for the 19-protein panel, the 15-protein panel, the 16-protein panel (purely data driven), the 10-protein panel (subset of 16 proteins that is also available in UK Biobank data) and NEFL alone. Red dots indicate overestimation and blue dots underestimation. The gray region represents an absolute difference of <1 year between observed and predicted values. Cor, Pearson’s correlation coefficient; RMSE, root mean square error (average error between predicted and observed).

We found that logistic regression (LR) models achieved performance comparable to the other top-performing models, while requiring only a relatively small number of proteins. Using stepwise LR, we then identified, for each timeframe, the model with the highest AUC of the receiver operating characteristic (ROC) curve. All subsequent results reported in this section are derived from the LR method with fivefold crossvalidation. The peak predictive performance on the validation set for each timeframe was: for 6-month prediction, 20 proteins, AUC = 0.945; for 1-year prediction, 11 proteins, AUC = 0.963; for 2-year prediction, 24 proteins, AUC = 0.939; for 3-year prediction, 34 proteins, AUC = 0.903; and for 5-year prediction, 25 proteins, AUC = 0.897 (Extended Data Table 3). By comparison, the respective validation AUCs for the single protein model that included NEFL alone ranged from 0.67 to 0.82 across the five timeframes (Fig. 4a,b). NEFL, not surprisingly, consistently appeared as the strongest single predictive marker across all five timeframes (Fig. 4a). It is interesting that two other proteins, dual specificity phosphatase 29 (DUSP29) and calcitonin-related polypeptide α (CALCA), also appeared in the models across five and four timeframes, respectively (Fig. 4c). The 77 unique proteins that were identified in these models and their overlap across the five timeframes, as well as the respective AUCs, are summarized in Fig. 4a and Extended Data Table 3.

From these 77 proteins, we first selected a subset to form a core panel of proteins, using a data-driven approach supplemented by expert curation (further described in Methods, step 4). We considered the ranking of each protein for phenoconversion prediction within each timeframe, favoring those ranked more highly, and sought to ensure a balance of biomarkers between those that were predictive of phenoconversion across timeframes and those predictive only for a single timeframe (Extended Data Table 3). This led to the compilation of a 19-protein biomarker panel that included the following: NEFL, DUSP29, CALCA, NEB, NGRN, MYL11, NOS1, MYH1, TNNC1, DTNB, MEGF10, SYNM, APOA4, ART3, MYL3, EPHA1, EDA2R, TTN and ACTN2. Procedures for protein selection are summarized in Extended Data Fig. 3 (right panel). Moreover, we explored a purely data-driven approach using greedy forward selection (further described in Methods, step 4), which revealed a panel of 16 proteins, including 7 (NEFL, DUSP29, CALCA, NGRN, NOS1, MYH1 and EPHA1) overlapping with the 19-protein panel (Extended Data Table 1). As the 19-protein and 16-protein panels were broadly consistent in predictive performance, we favored the 19-protein panel given that the 16-protein panel included some proteins with unclear biological relevance to ALS.

**Extended Data Fig. 4:**
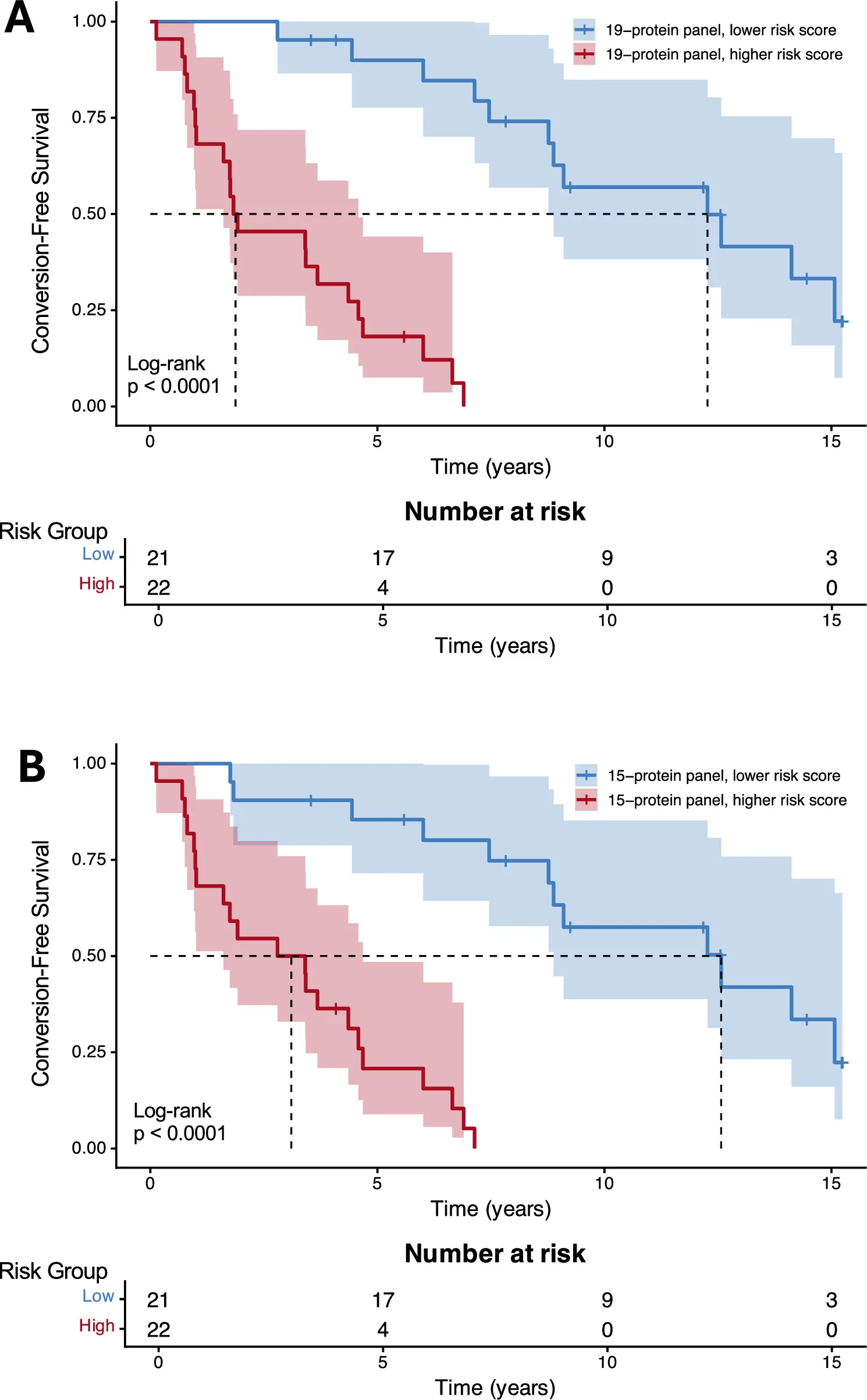
Phenoconversion-free survival based on core protein panel risk scores at baseline. Kaplan-Meier curves showing phenoconversion-free survival in the discovery cohort, by baseline risk groups (median split) for (**A**) the 19-protein panel (median conversion-free survival: 1.9 vs. 12.3 years for high- vs. low-risk groups; log-rank p = 4.48E-09), and (**B**) the 15-protein panel (median 3.1 vs. 12.6 years; log-rank p = 4.59E-08). Individuals were classified into high- and low-risk groups based on risk scores derived from LASSO Cox regression. Shaded areas represent 95% confidence intervals. Tick marks indicate censored observations.

In predicting phenoconversion, the 19-protein panel outperformed NEFL alone (Fig. 4b), which in turn outperformed any other single protein for any timeframe (Fig. 4a), underscoring the need for a multi-biomarker approach to forecasting phenoconversion across both short-term and intermediate-term timeframes. There was, however, one exception: for predicting phenoconversion within 0.5 year, the 19-protein panel (which also included NEFL) and NEFL alone performed comparably, suggesting that the dramatic rise in NEFL in the months immediately before phenoconversion may be sufficient for short-term prediction (Fig. 4a,b). The specific protein sets selected for each timeframe and their overlap across models are summarized in Extended Data Table 3 and illustrated in Fig. 4c, revealing shared and distinct biomarkers associated with different time periods before phenoconversion. Moreover, for comparison, Fig. 4b also shows the AUC of a 15-protein subset of these 19 proteins, selected based on their availability in the UK Biobank data, which we used for replication analysis (see ‘UK Biobank replication’ below; Extended Data Table 1). In addition, to further illustrate the prognostic performance of the 19-protein and 15-protein panels, we constructed partially penalized, LASSO, Cox proportional hazards models using baseline protein levels to predict time to phenoconversion in the discovery cohort; phenoconversion-free survival was significantly different between those with LASSO-derived risk scores above versus below the median (*P* < 0.001; Extended Data Fig. 4).

As AUC is a global summary measure of protein panel performance, we determined, for each protein panel and across the five phenoconversion timeframe horizons, the sensitivity and specificity at the threshold that maximized Youden’s index. For NEFL alone, sensitivity and specificity ranged between 61% and 81% and between 84% and 93%, respectively, whereas for the 19-protein panel, sensitivity and specificity ranged between 76% and 95% and between 76% and 95%, respectively (Extended Data Table 4).

### Phenoconversion timing estimation

In addition to predicting binary phenoconversion status within specified timeframes, we applied a linear truncation regression model to estimate time to phenoconversion among phenoconverters. Age, sex and genotype group were included as covariates. For the 19-protein panel, the correlation (cor) between estimated and actual years to phenoconversion was 0.79, with a mean absolute error (average absolute difference between predicted and observed) (MAE) of 1.62 years (Fig. 4d). A 15-protein panel—to facilitate replication analysis using UK Biobank data (see ‘UK Biobank replication’ below)—yielded comparable performance (Fig. 4d; cor = 0.76, MAE = 1.72), as did the fully data-driven 16-protein panel (Fig. 4d; cor = 0.82, MAE = 1.60) and the 10-protein subset to facilitate replication analysis (Fig. 4d; cor = 0.73, MAE = 1.86). Compared to NEFL alone (Fig. 4d; cor = 0.48, MAE = 2.37), the 19-protein panel showed significant improvement (likelihood ratio test, *P* < 0.0001).

### UK Biobank replication

**Fig. 5:**
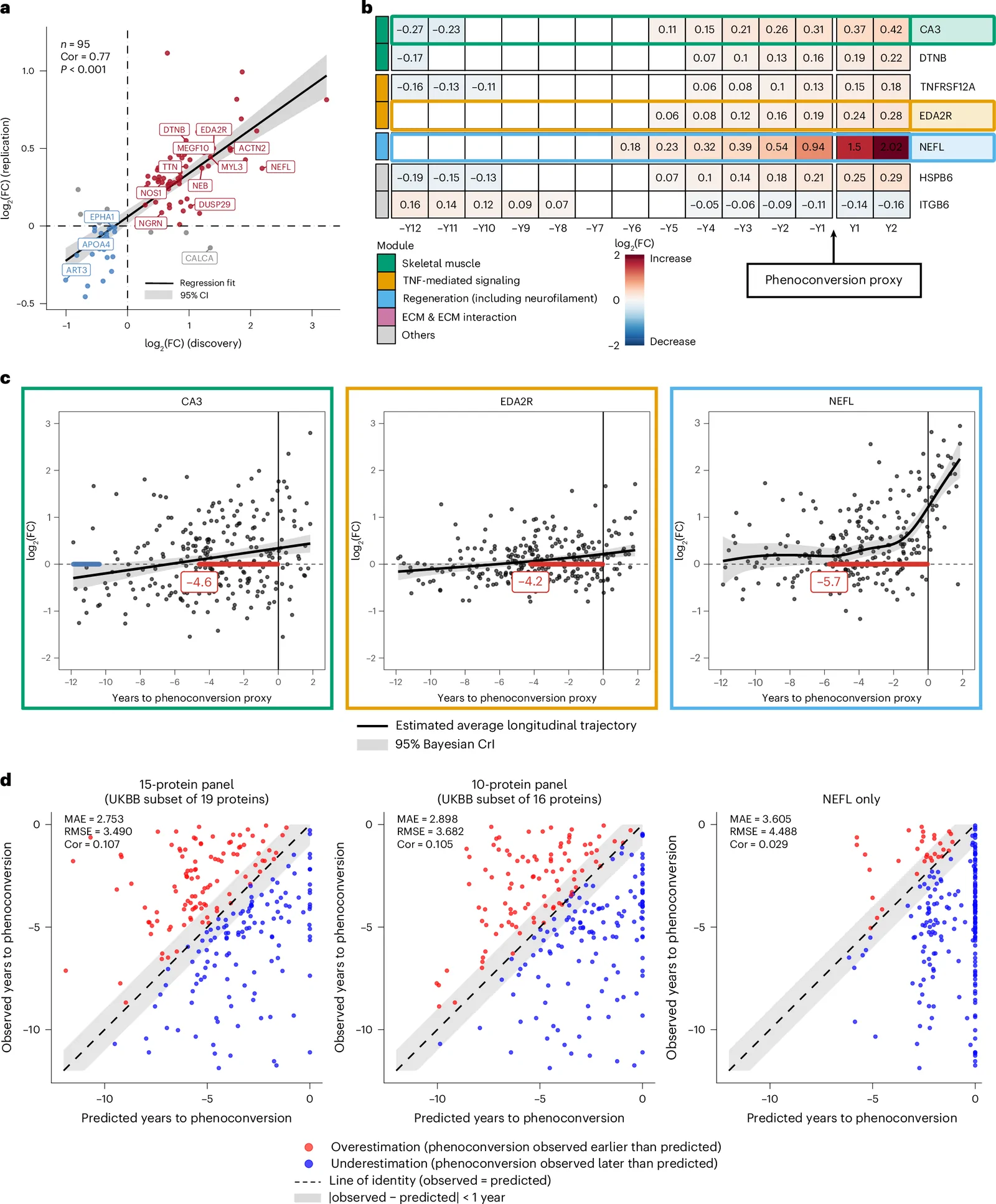
UK Biobank replication. **a**, Scatterplot showing high correlation between discovery and UK Biobank replication cohorts, for proteins that were differentially upregulated and downregulated in clinically manifest ALS versus healthy controls. Two-sided *P* = 6.69 × 10^−20^. The black line represents the fitted linear regression line, with the gray region denoting the corresponding 95% CI. **b**, Pseudo-longitudinal patterns (constructed using cross-sectional data) of seven proteins with relative abundance that deviated significantly from controls. This heatmap included data from the phenoconverter and pre-hospitalization groups. The number in each cell represents the average relative protein abundance at the indicated time interval, with negative and positive time on the *x* axis indicating, respectively, before and after the approximated time of phenoconversion. Empty cells denote time intervals where differences, compared to controls, were not statistically significant. The color scale reflects the direction and magnitude of change, with warmer colors (red) indicating increased concentrations relative to controls and cooler colors (blue) decreased concentration relative to controls based on the first layer of generalized additive model (GAM). Proteins are grouped by functional modules (Fig. 1d), as indicated by the color bar in the first column. **c**, Visual depiction of pseudo-longitudinal trajectories of proteins of interest (CA3, EDA2R and NEFL), relative to sex-matched and age-matched controls by first-layer GAM. Horizontal red and blue lines indicate the time periods during which protein levels of phenoconverters were, respectively, significantly higher (log_2_(FC) > 0) or lower (log_2_(FC) < 0) compared to controls. The gray shaded areas denote the 95% Bayesian CrI around the fitted mean trend for the relative protein abundance. **d**, Scatterplots illustrating the relationship between observed and estimated (or predicted) time to phenoconversion for the 15-protein panel, 10-protein panel and NEFL alone in UK Biobank data. The gray region represents an absolute difference of <1 year between observed and predicted values.

**Extended Data Fig. 5:**
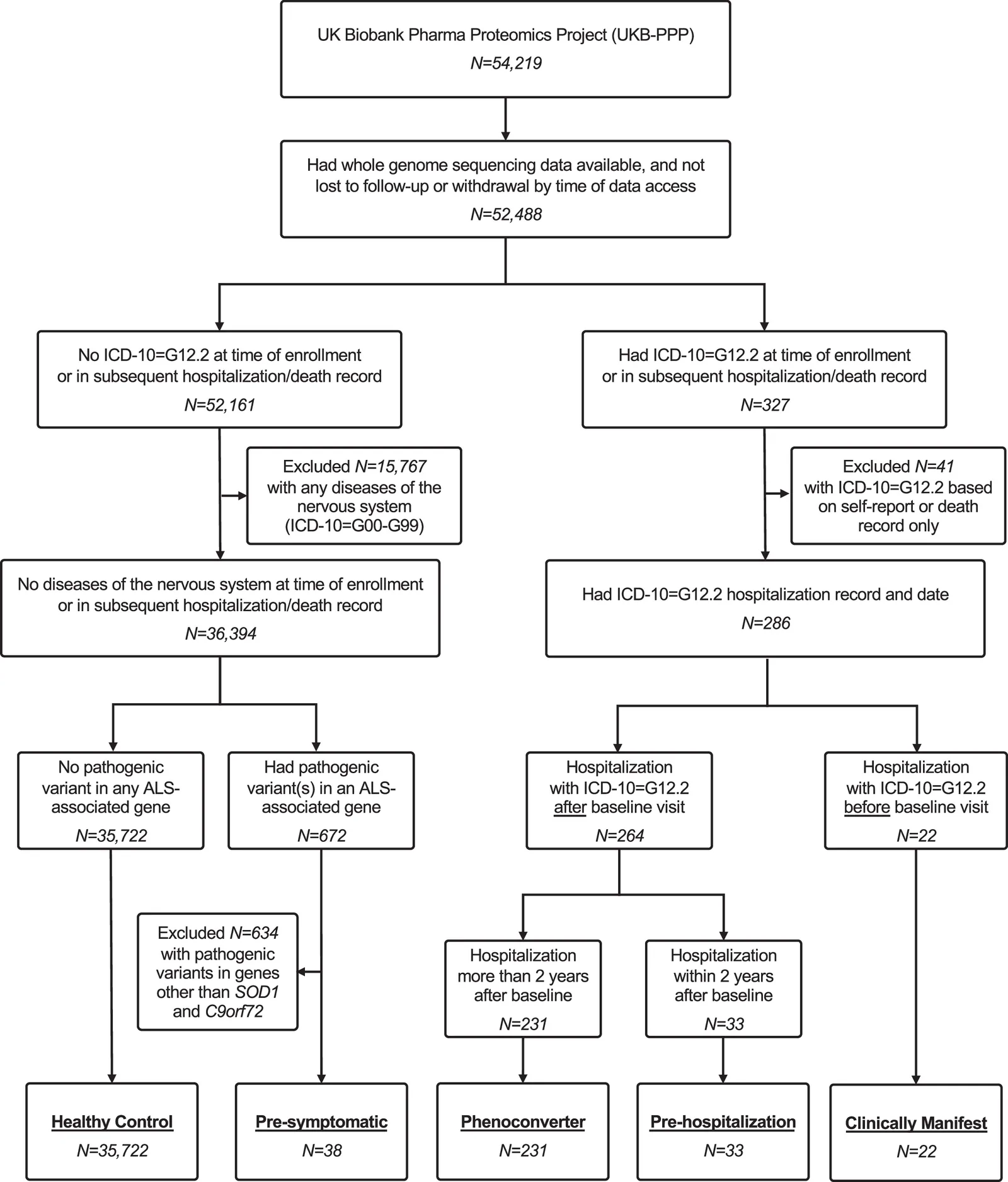
Subject selection process for replication cohort. Flowchart explicating the identification of healthy controls, pre-symptomatic carriers, phenoconverters, pre-hospitalization individuals, and those with clinically manifest ALS from UK Biobank data. Healthy controls were those with no records of nervous systems diseases and no pathogenic variant in an ALS-associated gene. Pre-symptomatic carriers were those with an *SOD1* pathogenic variant or *C9orf72* expansion of > 100 repeats, and no hospitalization or death record with an ICD-10 code of G12.2. Phenoconverters were those with G12.2 hospitalization date more than 2 years after UK Biobank baseline blood draw; since case identification for the replication cohort relied primarily on hospitalization records, we used 2 years prior to G12.2 hospitalization date as a proxy for phenoconversion date. Pre-hospitalization individuals were those whose first G12.2 hospitalization occurred within 2 years of the baseline blood draw. The clinically manifest ALS group included those with G12.2 hospitalization record prior to baseline blood draw.

For replication analyses, we utilized data from the UK Biobank Pharma Proteomics Project (UKB-PPP). Using inclusion and exclusion criteria that yielded participants comparable to the four groups in the discovery cohort, we identified from UKB-PPP *n* = 35,722 healthy controls, *n* = 38 pre-symptomatic *SOD1* and *C9orf72* carriers, *n* = 231 phenoconverters and *n* = 22 cases of clinically manifest ALS for inclusion in the replication cohort, as well as another *n* = 33 who comprised the pre-hospitalization group (Table 1 and Extended Data Fig. 5; see Methods for additional details). Notwithstanding some key differences between the two cohorts—proteomic data from UKB-PPP were cross-sectional and included 2,925 proteins (Olink Explore 3072), whereas our data were longitudinal and included 5,440 proteins (Olink Explore HT)—the top disease state biomarkers identified in the discovery and replication cohorts largely overlapped (Fig. 5a). By comparing phenoconverters to age- and sex-matched controls, we observed in the replication cohort similar temporal patterns to those in the discovery cohort for a subset of proteins: NEFL, EDA2R, TNFRSF12A, CA3, DTNB, HSPB6 and ITGB6 (Fig. 5b). Fifteen of the 19 core proteins, as well as 10 of the 16 purely data-driven proteins, identified in the discovery cohort were included in Olink Explore 3072, thereby constituting respectively a 15-protein and a 10-protein replication panel.

The trajectories of CA3, NEFL and EDA2R are illustrated in Fig. 5c. Importantly, these trajectories in the replication cohort are only pseudo-longitudinal, because they are compiled from cross-sectional data. As the timing of symptom onset (that is, phenoconversion) is not available in UKB-PPP, we approximated it to be 2 years before hospitalization with a relevant *International Classification of Disease*, 10th revision (ICD-10)^24^ code; this decision was informed by the clinical experience of UK-based ALS neurologists (personal communication, J.C.-K. and A.M.). Using truncated regression to estimate years from time of plasma collection to (approximated) phenoconversion, the 15-protein replication panel achieved the best performance (MAE = 2.75 years; Fig. 5d), outperforming both the 10-protein replication panel (MAE = 2.90 years) and NEFL alone (MAE = 3.61 years). Notably, in the discovery cohort, the 15-protein and 10-protein panels also outperformed NEFL alone (likelihood ratio test, *P* < 0.001).

Although a 4-year approximation window yielded better predictions of time to phenoconversion in subsequent sensitivity analyses performed across a range of approximation windows (that is, by approximating the time of phenoconversion to be 4, 3, 2, 1 or 0 years before hospitalization), we retained the 2-year approximation in the primary analysis because, as noted above, this was identified as most clinically appropriate. As shown in Extended Data Table 5, across all approximation windows, the 15-protein replication panel performed better (that is, had the lowest MAE values) than both the 10-protein replication panel and NEFL alone.

## Discussion

This study is the first to map the longitudinal trajectory of an array of protein biomarkers across the course of ALS disease progression, from pre-symptomatic through clinically manifest stages of disease. Prior studies have relied on cross-sectional proteomic measures^25,26^, made assumptions about the timing of phenoconversion^25,26^ or inferred pre-symptomatic biomarker trajectories based on data from pre-symptomatic and clinically manifest individuals rather than phenoconverters^17^. By contrast, our findings derive from longitudinal proteomic data from the largest cohort of phenoconverters evaluated both before and after the emergence of clinically manifest disease and with the timing of phenoconversion rigorously determined. In univariate analyses, we found that the concentration of 92 proteins deviated from age- and sex-matched controls before phenoconversion, with skeletal muscle, the ECM, neurofilament and regeneration, as well as positive regulation of TNF signaling, among the biological pathways most impacted. In addition to shedding light on the biology of pre-symptomatic disease, we have identified a 19-protein panel that predicts the event of phenoconversion across a range of time horizons from 0.5 year to 5 years, with crossvalidated AUCs of 0.80–0.89. Critically, these AUCs are not artificially inflated by comparing phenoconverters to healthy controls—a group that is more readily distinguishable from phenoconverters but would not be suitable as the comparison group for addressing clinical questions. Rather, these AUCs reflect the real-world challenge of differentiating phenoconverters from pre-symptomatic carriers who do not phenoconvert within the specified timeframe. Given that the present study included only a small (and selected) subset of pre-symptomatic carriers from Pre-fALS, we recognize the need to replicate these findings in a broader group. Nevertheless, these findings build on our prior observation of NfL’s utility as a susceptibility/risk biomarker predicting phenoconversion to clinically manifest ALS. Importantly, although NfL alone may be sufficient over the short term among carriers of highly penetrant *SOD1* variants associated with rapidly progressive disease, additional susceptibility/risk biomarkers are needed for longer-range prediction and for those with less aggressive forms of disease^7,11,12,16^. Moreover, although Cox model with time-dependent covariates did not yield informative results, Cox model utilizing baseline protein biomarker levels was informative—and this model is arguably more relevant to the eligibility criteria for future disease prevention trials.

We have also made meaningful inroads into the challenge of estimating the time to phenoconversion among pathogenic variant carriers. Before this study, with the exception of NfL in a subset of *SOD1* pathogenic variant carriers, we had limited ability to predict the timing of phenoconversion. In this study, however, using truncation models and our 19-protein panel (which includes NEFL) among phenoconverters, the only group in whom the timing of phenoconversion is definitively known, we were able to estimate years to phenoconversion with an MAE of 1.6 years. Assuming that the predictive value of this protein panel, when assayed through traditional immunoassays, is confirmed, this MAE represents a considerable advancement with practical implications and provides a temporal anchor for interpretating data from carriers who have not phenoconverted. For example, a prevention trial that aims to enroll individuals with an estimated time to phenoconversion of 2 years (or less), based on this protein panel, would require a follow-up duration of ~3.6 years, which is eminently feasible, as evidenced by experience from the ATLAS trial^13^.

In constructing a multi-protein panel to serve as a susceptibility or risk biomarker, we considered the pros and cons of adopting a purely data-driven approach versus a supervised data-driven approach augmented by expert curation. Although the former is less biased and may be more likely to maximize predictive performance, this approach is also susceptible to overfitting, poor reproducibility and limited biological interpretability. On the other hand, the latter (which is the approach that we have taken) risks reinforcing established ideas and may have lower predictive power. However, it benefits from yielding a result that is biologically more plausible, interpretable and with better reproducibility^27^. Ultimately the two approaches yielded comparable performances in phenoconversion prediction, although some of the proteins in the purely data-driven 16-protein panel are less biologically interpretable. Moreover, 7 of the proteins included in our 19-protein panel were among those highlighted by a recent publication as contributing to a risk score proposed for the diagnosis of ALS^25^.

We recognize the intrinsic bias of the targeted Olink platform, in which well-known, higher abundance, extracellular proteins are overrepresented. Nevertheless, our results are consistent with prior studies in identifying NEFL as one of the most differentially regulated proteins when comparing those with clinically manifest ALS to healthy controls. Of all the proteins examined, NEFL is also the single most useful biofluid-based biomarker of pre-symptomatic disease. However, the largest number of proteins identified as differentially regulated between ALS and controls, as well as those that change pre-symptomatically and contribute to predicting the timing of phenoconversion, are not neuronal; rather, they reflect the biology of skeletal muscle (consistent with a prior study that reported elevated creatine kinase levels before onset of weakness in eight patients with sporadic ALS^28^). Specifically, many of these are structural components of skeletal muscle: MYH1 and MY11 (highly expressed in fast-twitch fibers); MYL3, CRSP3, ANKRD2 and TNNC1 (slow-twitch); as well as TTN, NEB, SYNM and ACTN2 (sarcomeric). Several, on the other hand, reflect cellular processes related to skeletal muscle atrophy (DUSP29, EDA2R, MEGF10 and CALCA). DUSP29 is noteworthy because it is transcriptionally activated in denervation-induced atrophy, attenuating the ERK1 and ERK2 branch of the MAP kinase signaling pathway and inhibiting muscle cell differentiation^29^; DUSP29 was also identified as a pre-symptomatic marker based on SomaScan data in the EPIC cohort^26^. Similarly, EDA2R, a member of the TNF receptor superfamily, has emerged as an important mediator of skeletal muscle atrophy^30^. A loss of NOS1, which inhibits FOXO3, a transcription factor that induces proteolytic and autophagic pathways, is an early event in skeletal muscle atrophy^31^. MEGF10 is essential for skeletal muscle development and maintenance, with deficiency known to result in loss of muscle mass^32^. Moreover, CALCA, which is released from motor neurons and has a role in inhibiting autophagic lysosomal proteolysis, likely reflects an anabolic response to denervation-induced atrophy. Whereas the observed increase in MEGF10 and CALCA likely reflects a compensatory response to neurogenic atrophy, the increase in EDA2R and DUSP29 is unexpected and may reflect a role in driving or exacerbating muscle atrophy. The observation that CALCA is elevated in ALS versus controls and among pathogenic variant carriers before ALS phenoconversion is also of interest, given the studies that have identified increased expression of calcitonin gene-related peptide as a marker of motor neuron vulnerability in the SOD1^G93A^ mouse^33^. Notwithstanding the foregoing, we do recognize the inherent limitations of drawing biological inferences based on blood proteomic data.

Major strengths of this study include the use of longitudinal proteomic data and detailed information about the timing of phenoconversion, in the largest available series of phenoconverters with such data available. A limitation of our study, however, is the lack of an independent, but comparable, cohort for replication. Nevertheless, we were able to utilize Olink data from the UK Biobank study, which includes whole-genome sequencing and proteomic data from participants who subsequently developed (mostly sporadic) ALS. A key challenge was that the exact timing of phenoconversion is not available in UK Biobank data. Instead, we relied on ICD-10 codes for motor neuron disease (MND, G12.2) at the time of hospitalization. As hospitalization is not an early event in the course of ALS, these data do not readily lend themselves to replication of an analysis focused on phenoconversion. To partially mitigate this issue, we assumed symptom onset to be 2 years before hospitalization with an associated ICD-10 code of G12.2 and used this as the proxy timepoint of phenoconversion. Despite the imperfections of this approach, we were able to replicate the findings of NEFL, EDA2R, CA3, TNFRSF12A, DTNB, ITGB6 and HSPB6 as pre-symptomatic biomarkers. We also showed that a multi-protein panel improved estimates of the predicted time to phenoconversion above and beyond what can be accomplished using NEFL alone—a finding that was further confirmed in our sensitivity analyses using a range of approximation windows for the timing of ALS symptom onset relative to hospitalization. We are, however, cautious not to overinterpret the MAE values in the replication cohort, because they are based on imputed time of symptom onset. That the results from our discovery cohort were only partially replicated in the UK Biobank cohort is likely due in part to the limitations of relying on cross-sectional proteomic data and very imprecise proxies of the timing of phenoconversion in the latter, as well as differences in proteomic platforms between the two cohorts. As such, we viewed these replication analyses as providing qualitative and directional support for, rather than quantitative verification of, the results in discovery cohort.

In modeling the pre-symptomatic trajectory of protein biomarkers, we have utilized data from phenoconverters with longitudinal measurements both before and after phenoconversion, as well as data from those with clinically manifest ALS. It is noteworthy that the number of protein biomarker candidates and estimates of pre-symptomatic trajectories differ somewhat when, instead, relying solely on longitudinal data from phenoconverters pre-conversion and post-conversion, or when using only pre-conversion data from phenoconverters. Importantly, models that rely solely on data from pre-symptomatic carriers who have not yet phenoconverted (and in whom the timing of phenoconversion is unknown) or data solely from those with clinically manifest ALS (in whom the timing of phenoconversion is retrospectively estimated) are inherently less robust, because they lack observed or measured timing of phenoconversion.

As other ongoing pre-symptomatic studies^34–37^ accrue larger numbers of phenoconverters and more longitudinal visits and large-scale proteomic analyses become more accessible, it will be important to more fully replicate our findings. As data from a larger number of phenoconverters become available in the Pre-fALS stiudy, it will also be important to interrogate the effects of genotype and to understand the extent to which the pre-symptomatic trajectory of protein biomarkers differs based on the underlying genetic cause of disease. Moreover, practical utilization of a panel of proteins as a susceptibility or risk biomarker will benefit from immunoassay validation of leading candidates, as well as demonstrate that the results of such immunoassays hold sufficient predictive value for use as eligibility criteria for trial enrollment or randomization. Meanwhile, the results reported here are immediately relevant to the design of future ALS prevention studies. Specifically, clinical trials that aim to prevent phenoconversion to clinically manifest ALS/FTD will need to enrich the study population for those at greatest short-term risk of the primary outcome measure that will be used to quantify treatment effect. As such, our identification of a panel of proteins that reliably predict the occurrence and timing of phenoconversion represents an important advance in furthering the ultimate goal of ALS prevention.

## Methods

Overall study design and statistical methods are summarized in Extended Data Fig. 1. The discovery cohort comprised participants from the parent studies at the University of Miami (described below). The replication cohort was drawn from the UK Biobank. As previously described, we regard the pre-symptomatic stage of disease to be from the onset of the underlying disease (biologically defined) to phenoconversion^8,9,38,39^. Throughout this manuscript we use the nonitalicized gene name to refer to the protein product. We adopted this convention for ease of visualization and to enable comparability to the published literature.

### Parent studies: cohort descriptions, ethics approvals, sample collection and processing

This proteomic study included participants from three parent studies: the Pre-fALS study, the Clinical Research in ALS (CRiALS) Biomarker study and the CReATe Consortium Phenotype–Genotype–Biomarker (PGB1) study. Details of the Pre-fALS study (accession no. NCT00317616) have previously been described^11,12,18,20,21^. Briefly, Pre-fALS is a single-center study that recruits, from across North America, individuals who are carriers of any ALS (or ALS/FTD)-associated pathogenic variant and who, at the time of enrollment, are clinically pre-symptomatic for ALS and FTD (see Supplementaty Information for additional details). The CRiALS (accession no. NCT00136500) Biomarker study is a companion study to Pre-fALS, serving to recruit both healthy controls and patients with clinically manifest ALS, to aid the interpretation of pre-symptomatic data from Pre-fALS study. The CRiALS Biomarker study procedures and clinical assessments mirror those used in Pre-fALS. Additional participants with clinically manifest ALS were drawn from the CReATe PGB1 study (accession no. NCT02327845), a multi-center natural history study of individuals with ALS and related disorders, in which participants were evaluated serially to acquire longitudinal phenotypic data and biospecimens. PGB1 study participants included in this report were all from the University of Miami site.

All three studies were approved by the University of Miami institutional review board (IRB), which also serves as the single IRB of record for the CReATe Consortium, and all study participants provided written informed consent. The University of Miami IRB operates under the umbrella of the University of Miami Human Subjects Research Office (FWA00002247).

Plasma samples were collected, processed and stored according to strict standard operating procedures. Briefly, blood was collected in K2 EDTA tubes, centrifuged at 1,750*g* for 10 min and 4 °C and aliquoted for storage at −80 °C.

### Experimental design: participant and sample selection and Olink plate assignment

An experiment of 516 plasma samples was planned, with a focus on phenoconverters, but also including pre-symptomatic pathogenic variant carriers who have not developed ALS as well as both healthy controls and patients with clinically manifest ALS.

All Pre-fALS phenoconverters with at least two plasma collections at the time of sample selection were included. A small number of pre-symptomatic participants thought to be at higher likelihood of phenoconversion in the near future, based on clinical or biomarker data, were also selectively included. The rationale for their selection was the potential to increase the number of phenoconverters by the time of data analysis (notwithstanding that this particular subset of pre-symptomatic carriers might bias some of the results toward the null). Indeed, two of them phenoconverted before final data analyses (data cutoff: January 2025) and were included in this report as phenoconverters. Controls with known comorbidities were excluded. If a control participant had more than three plasma collections, the first and last collections along with a collection near the mid-point of study follow-up were included (exception: *n* = 1 control had four collections included). The clinically manifest group included those with at least three plasma collections (exceptions: *n* = 2 each had just two samples with sufficient volume available) and were broadly representative of patients at different stages of the disease. Plasma samples collected at the time of known or possible exposure to antisense oligonucleotides (through clinical administration or clinical trials) were excluded. The groups were age- and sex-matched as much as possible.

In the Olink experiment, the 516 plasma samples (40 µl each) were run on 6 plates of 86 samples each. Longitudinal samples from the same participant were included on the same plate. In addition, plate assignment was devised to achieve a balanced design, with similar distributions of participant group (control, pre-symptomatic, phenoconverter and clinically manifest), age and sex (based on self-report) on each of the six plates.

### Proximity extension assay, library preparation and next-generation sequencing

Olink library preparation and sequencing were performed using the Explore proximity extension assay (PEA) technology and the Explore HT protein biomarker panel. PEA was performed as per the proteomic method previously described^40^. Briefly, the PEA technology employs high-multiplex matched pairs of antibodies, each conjugated to a unique DNA oligonucleotide. These antibodies were incubated with their respective target proteins, facilitating specific binding. Upon hybridization of antibodies, DNA polymerase-mediated extension generated a unique DNA barcode for each antibody–target interaction, wherever both specific antibodies recognized the same protein. The resulting DNA barcodes were amplified using PCR, incorporating unique sample indexes to enable multiplexing of samples. After amplification, Olink libraries were purified using Agencourt AMPure XP magnetic beads to remove unwanted PCR byproducts. The quality of the purified libraries was assessed using an Agilent TapeStation system to ensure proper fragment size distribution and integrity. Each assay has been extensively validated for limit of detection, measurement ranges, precision, reproducibility and specificity, as previously described^41^. The prepared Olink libraries were sequenced using S4 flow cells on the Illumina’s NovaSeq 6000 platform. Data underwent QC checks to ensure sequencing accuracy and integrity.

### Normalizing protein expression

Olink-normalized protein expression (NPX) represents the relative protein concentration units on a log_2_ scale. The values were calculated from the number of sequencing reads matching the reference Olink protein barcodes. Data were normalized to internal extension controls, spiked in during the sample preparation and then log_2_-transformed. As the study involved more than a single plate of samples, the data for the whole cohort were then normalized to remove plate-to-plate variations. Briefly, for each plate and assay, the plate-specific median value was calculated and then the plate-specific median subtracted from every sample of the plate; this centralized the median to 0. Illumina raw data were converted to counts using Olink’s NGS2counts pipeline and NPX calculations, and QC analysis was performed with Olink’s NPX Explore HT software.

### Additional QC procedures

A two-stage filtering strategy was employed: First, only proteins with Olink’s SampleQC and AssayQC metrics both annotated as ‘PASS’ were retained, whereas those with either metric annotated as ‘NA’, ‘WARN’ or ‘FAIL’ were excluded. Second, proteins with excessive missingness or low-read measurements were excluded. The dataset was reduced from 5,440 proteins to 5,298 proteins.

### Statistical analysis

We applied machine learning methods to predict the occurrence and timing of phenoconversion. Our method for discovery progressed through five steps: differentially expressed protein selection, temporal dynamics protein modeling, time-to-phenoconversion Cox modeling, phenoconversion event prediction and phenoconversion timing estimation. All statistical tests were two sided and all analyses were performed in R 4.4.0.

#### Step 1. Differentially expressed protein selection

##### Step 1a. Differentially regulated protein identification

The goal in this step was to select a subset of proteins differentially expressed between clinically manifest ALS and healthy controls (that is, disease state biomarkers) from the ~5,300 in the Olink Explore HT panel for downstream analysis. This step was based on the rationale that proteins differentially expressed between patients with clinically manifest ALS and healthy controls are likely ALS related and would be strong candidates to predict phenoconversion among pathogenic variant carriers. To ensure no overlap between samples used in this step and those used to build prediction models, we included in this step only study participants from the clinically manifest (*n* = 35) and healthy control (*n* = 59) groups, each with Olink data from 126 visits (Table 1). As all but 21 controls contributed multiple samples, we employed a mixed-effects model with a random intercept to account for within-person correlation. We also included age at sample collection, sex and genotype group (*SOD1* A4V, *SOD1* non-A4V, *C9orf72* and other pathogenic variants) as covariates. The Benjamini–Hochberg (BH) procedure was applied to control the FDR^42^ at a significance threshold of 0.05.

##### Step 1b. Biological implications

Pathway enrichment analysis was performed using the ‘gost’ function in the gprofiler2 package (v0.2.3), which provides functional profiling by mapping differentially expressed proteins to GO, *Kyoto Encyclopedia of Genes and Genomes*, Reactome and other databases^43^. Statistical significance was defined as an FDR-adjusted *P* value (*P*_adj_) < 0.05.

Protein–protein interaction (PPI) networks were constructed to investigate the functional connectivity among the differentially expressed proteins. Interactions were queried using the STRING database (v11.5), which integrates evidence from experimental data, computational prediction, co-expression, curated databases and literature mining^23^. To identify biologically coherent subnetworks, we applied Markov Cluster Algorithm clustering using STRING’s built-in implementation, with an inflation parameter set to 3 to control cluster granularity.

#### Step 2. Temporal dynamics protein modeling

To evaluate whether the differential abundance observed between patients with clinically manifest ALS and healthy controls can also be detected before phenoconversion, we modeled the longitudinal trajectories of the subset of proteins identified in step 1, using a two-layer GAMM framework^44^.

Details of the GAMM models are available in Supplementary Information. Briefly, in the first layer, we modeled the protein trajectory in healthy controls only and included sex as a fixed covariate, a random intercept to account for within-participant correlation, and a smooth function of age to capture nonlinear age-related changes in protein abundance. In the second layer, we modeled the relative protein abundance—defined as the deviation from the expected protein abundance in an age- and sex-matched healthy control based on the model in the first layer—over time, including also a random intercept and a smooth function to capture nonlinear protein trajectories. To assess the statistical significance of the disease effect in each model, we applied the BH procedure on smooth terms to control the FDR at a significance threshold of 0.05.

For each protein that exhibited a significant pre-symptomatic change, we determined the earliest time of this change by first constructing the pointwise 95% Bayesian credible interval (CrI) around the fitted curve of the longitudinal protein trajectory, with s.e. values obtained from the Bayesian posterior covariance matrix of the spline coefficients under REML estimation in the mgcv package^44^; these intervals have good frequentist across-the-function coverage properties^45^. We then identified the time period(s) during which this CrI excluded 0, indicating a significant difference from age- and sex-matched controls, and considered the start of this period to be the earliest time of pre-symptomatic change (see Supplementary Information for additional details).

In primary analyses, the models included data from both phenoconverters and those with clinically manifest ALS. For the latter group, reported onset of weakness was used as a proxy for phenoconversion. Secondarily, we repeated these models but included only data from phenoconverters (pre-conversion and post-conversion) or only pre-conversion visits from phenoconverters.

#### Step 3. Time-to-phenoconversion Cox regression

To assess the association between protein biomarker and subsequent phenoconversion, we performed time-to-event analyses using Cox proportional hazards models and baseline protein biomarker data from phenoconverters (that is, those who met phenoconversion endpoint) and pre-symptomatic individuals (that is, those who were censored). We first fitted univariate Cox models, including relative protein abundance as the predictor and adjusting for covariates (age, sex and genotype group). Multiple testing was controlled for using the BH procedure (FDR < 0.05). For multivariable prediction, we constructed partially penalized Cox models with LASSO regularization, penalizing only the protein variables while retaining clinical covariates without penalty. The optimal penalty parameter was selected using leave-one-out crossvalidation. The LASSO-derived risk score for each participant was calculated as a weighted sum of the selected proteins and covariates, using the regression coefficients estimated from the optimal penalized Cox model. The performance of different protein panels was evaluated by comparing those with high versus low risk score (median split) using Kaplan–Meier analysis and log-rank test.

To leverage longitudinal proteomic measurements, we additionally explored univariate and LASSO-penalized, time-dependent Cox models, in which protein abundance was treated as a time-varying covariate updated at each visit. Univariate models were evaluated using the BH procedure with FDR < 0.05 and LASSO penalization was applied to protein variables only, with clinical covariates retained unpenalized and the optimal penalty parameter selected by leave-one-out crossvalidation.

#### Step 4. Phenoconversion event prediction

Using the differentially expressed proteins (that is, disease state biomarkers) identified in step 1, this step aimed to build prediction models for whether a pre-symptomatic pathogenic variant carrier will phenoconvert to ALS within a certain timeframe (*T*), with *T* ranging from 0.5 years to 5 years. To enhance model robustness, we used absolute protein levels (NPX) rather than relative protein abundance in these prediction models. This choice eliminates the dependence on a reference control population for calibration and ensures applicability in clinical settings where repeated measurements or a similar control reference may not be available. Training data were drawn from the pre-symptomatic and phenoconverter groups, with each visit annotated as either ‘case’ or ‘control’, depending on, respectively, whether or not phenoconversion occurred within the specified time period after the visit. For the pre-symptomatic group, all visits with subsequent follow-up time longer than (or equal to) *T* were annotated as ‘controls’; the remaining visits were excluded because we do not know whether the participant might still phenoconvert within the *T-*year interval. For the phenoconverter group, all visits where phenoconversion occurred more than *T* years after the visit were considered to be ‘controls’, whereas all visits where phenoconversion occurred within *T* years of the visit were considered to be ‘cases’. Each phenoconverter’s first post-conversion visit was also included and labeled as a ‘case’, whereas all other post-conversion visits were excluded.

We evaluated seven machine learning approaches and decided on LR, with fivefold crossvalidation for model training and testing (see Supplementary Information for additional details).

We tested five different timeframes: *T* = 0.5, 1, 2, 3 and 5 years. For each timeframe, we first ran univariate LR for each protein individually, with covariates for sex, age (at sample collection) and genotype group. From the results, we identified the proteins that returned the best prediction measured by AUC of a ROC curve. Next, we performed a greedy stepwise LR with forward greedy search. Specifically, we progressively added, one at a time, proteins that maximized the incremental (or minimized the decremental) predictive performance among all remaining proteins. Performance was measured using the average AUC across the fivefold crossvalidation. The model with the best predictive performance in the testing set was selected as the designated model for that timeframe. We counted how many times each of the differentially expressed proteins from step 1 are included in the five designated models (one for each timeframe). Among the proteins that appeared at least once in the five models (Extended Data Table 3), we further filtered them based on their ranking, the timeframe(s) in which they appeared, as well as biological rationale to arrive at a small core panel of proteins as susceptibility/risk biomarkers (see Supplementary Information for additional details).

Similarly, and in parallel, we undertook a purely data-driven approach (that is, without expert curation), using greedy forward selection to identify a small panel of proteins that predicted phenoconversion across the different timeframes, based on their average crossvalidation AUCs across these timeframes.

To determine the optimal classification threshold of the resulting core protein panels, we used Youden’s index derived from the ROC curve. Specifically, for each model we identified the threshold that maximized Youden’s *J* statistic (sensitivity + specificity − 1), which represents the point that optimally balances sensitivity and specificity. This threshold was then used to predict whether an individual would phenoconvert within *T* years after the visit, classifying each individual as either a converter or a nonconverter.

To illustrate the association between the baseline risk score of multi-protein panels and phenoconversion-free survival, we employed partially penalized LASSO Cox analysis, as well as Kaplan–Meier analysis and log-rank test, as described in step 3 above.

#### Step 5. Phenoconversion timing estimation

Our goal in this step was to build disease progression models to estimate pre-symptomatic mutation carriers’ proximity to time of phenoconversion given the proteomic profile obtained from the individual’s most recent visit and baseline visit, similar to the approach employed in a recent study of familial FTD that utilized a Bayesian disease progression model^17^. For this step, we used the final protein panel identified in step 4. Instead of LR, we used a truncated regression model and included only data from the pre-conversion visits of phenoconverters, the only group in whom time to phenoconversion is known. To avoid the collinearity between age and time to phenoconversion, we used relative protein abundance from step 2 as input features. Given the longitudinal structure of the proteomics data, with repeated measurements collected from the same individuals over time, we used each participant’s baseline visit as an internal reference to account for interindividual variability in protein abundance. To model time to phenoconversion as a continuous outcome, we employed truncated regression and included sex and genotype group as covariates (see Supplementary Information for additional details).

The predicted and observed time to phenoconversions were compared and summarized using the RMSE, MAE and Pearson’s correlation. The performance of the multi-protein panel model was compared to the NEFL-only model using the likelihood ratio test to determine whether the multi-protein panel provided a significant improvement in phenoconversion timing estimation above and beyond NEFL.

### UKB replication

For replication analysis, we used data from the UK Biobank (UKB) cohort. UKB is a large-scale prospective study that recruited 503,317 participants aged 40–69 years between 2006 and 2010 across the UK^19,46^, with Olink Explore 3072 data available through the UKB-PPP for *n* = 54,219^47^. Data for the present analysis were accessed under application no. 847687.

The replication cohort consists of five participant groups (Table 1), four of which are similarly defined as in the discovery cohort: heathy control, pre-symptomatic, phenoconverter and clinically manifest. A fifth group (pre-hospitalization) was also included in the modeling of protein trajectories (see Supplementary Information for detailed description of the inclusion and exclusion criteria for each group).

From among the UKB-PPP participants, because we need genotype information to determine eligibility for the control versus the pre-symptomatic group, as well as to adjust for genotype in replication analyses (as is done in discovery analyses), we included only the *n* = 52,488 for whom whole-genome sequencing data were also available and who had neither withdrawn consent nor been lost to follow-up (datafield 190 in UKB), as of the time of data access in August 2025 (Extended Data Fig. 5).

For replication analysis, we applied the differential protein expression approach (as described in step 1 above) to the UKB-PPP cohort, using covariate-matched healthy controls for individuals in the clinically manifest group, with covariates including sex, age and genotype group. Of the 137 proteins identified in the discovery cohort, 95 were available in the UKB due to differences between the Olink Explore 3072 and the Olink Explore HT platforms. We plotted the log_2_(FC) of the overlapping proteins and assessed the correlation between discovery and replication cohorts via Pearson’s correlation. To analyze the temporal dynamics of proteins in the replication cohort, we applied two-layer GAMs instead of two-layer GAMMs, because the UKB data were cross-sectional rather than longitudinal. Accordingly, individual-level random effects were excluded from the models. In the first-layer GAM, relative protein abundance was modeled with a covariate sex and a smooth term of age. The second-layer GAM modeled relative protein abundance against a smooth term of proxy for time to phenoconversion. We then tested our phenoconversion timing estimation models trained on the discovery cohort to baseline protein expression levels in the replication cohort, with covariates including sex, age and genotype group.

### Reporting summary

Further information on research design is available in the Nature Portfolio Reporting Summary linked to this article.

## Online content

Any methods, additional references, Nature Portfolio reporting summaries, source data, extended data, supplementary information, acknowledgements, peer review information; details of author contributions and competing interests; and statements of data and code availability are available at https://doi.org/10.1038/s41591-026-04528-x.

## Supplementary information

Supplementary Information Supplementary Figs. 1 and 2. Detailed methods: (A) parent studies, (B) statistical analysis, (C) UK Biobank replication.

Reporting Summary

Supplementary Tables Supplementary Tables 1 and 2.

## Figures and Tables

**Extended Data Table 1:**
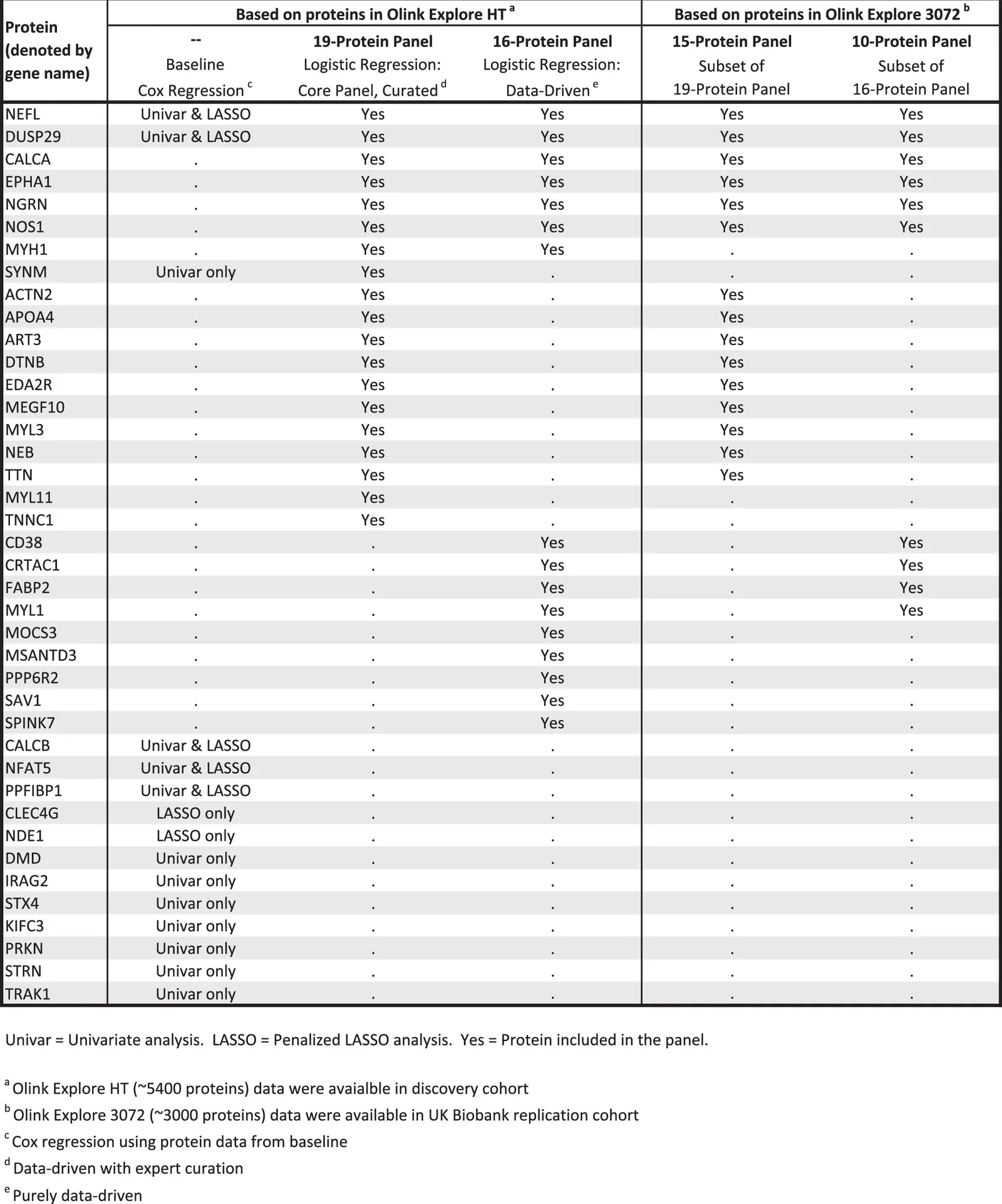
Protein candidates identified by different analytic approaches

**Extended Data Table 2:**
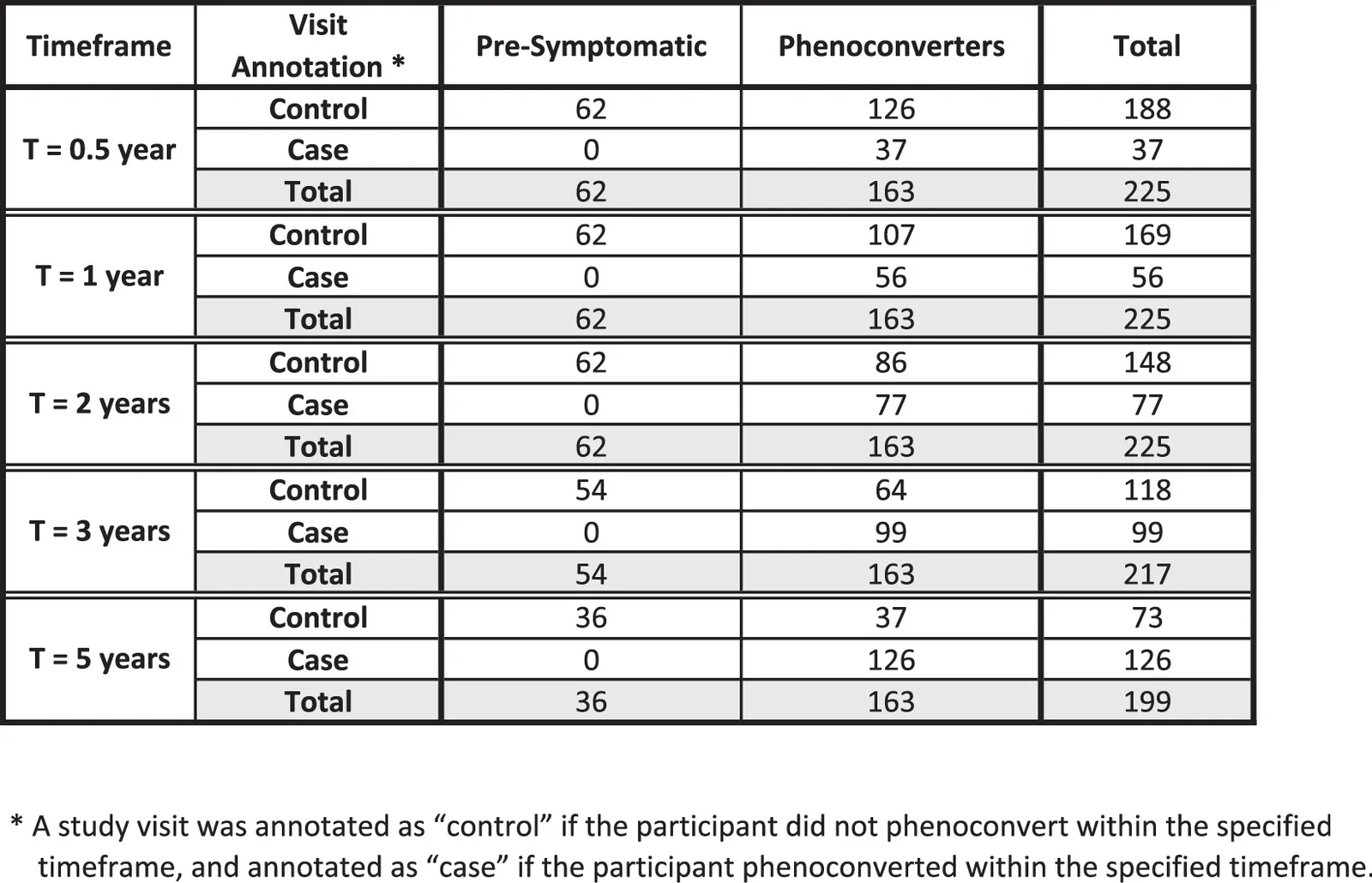
Number of visits annotated as cases vs. controls for each prediction interval

**Extended Data Table 3:**
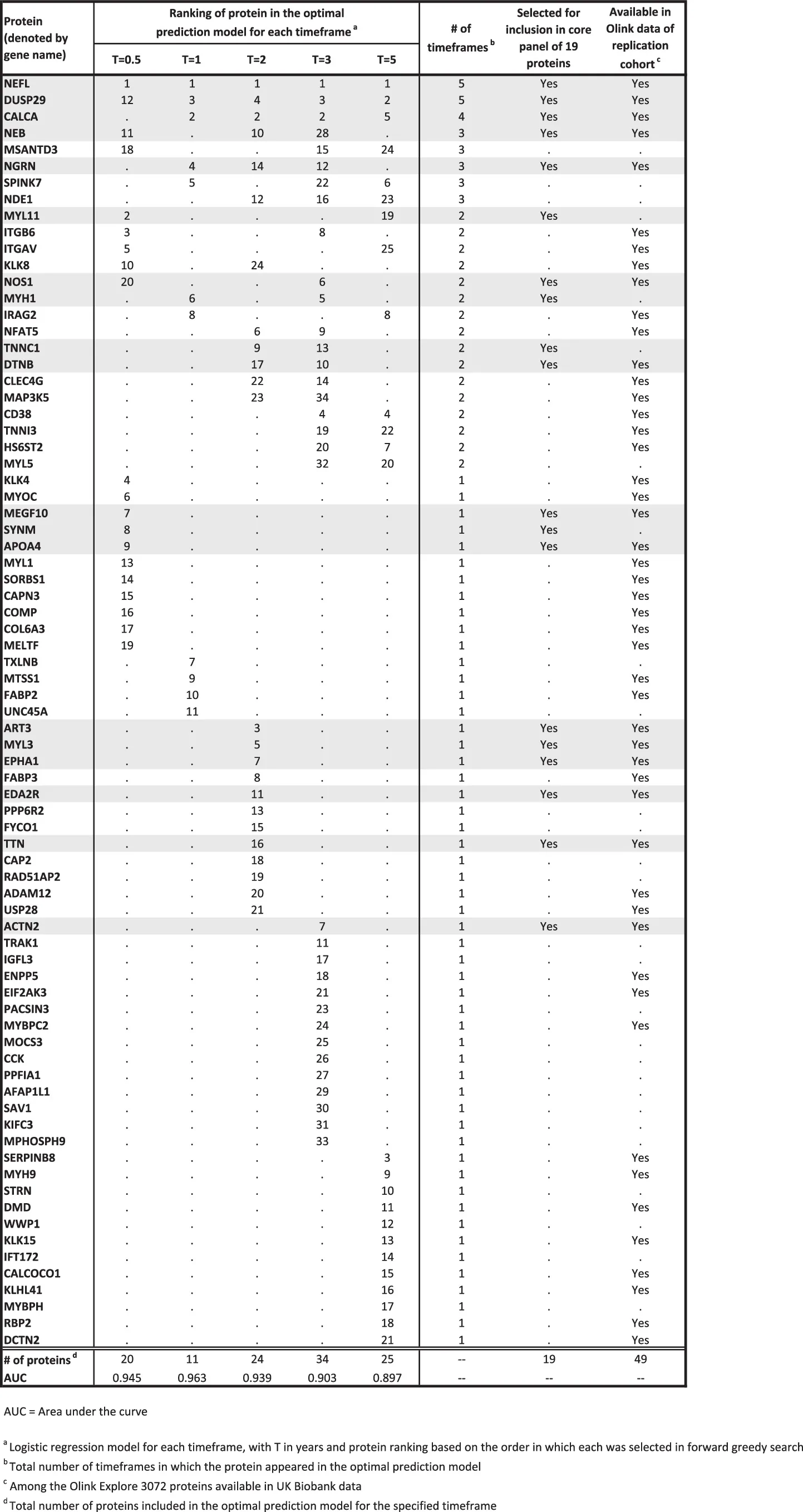
Protein selection based on phenoconversion event prediction across time horizons

**Extended Data Table 4:**
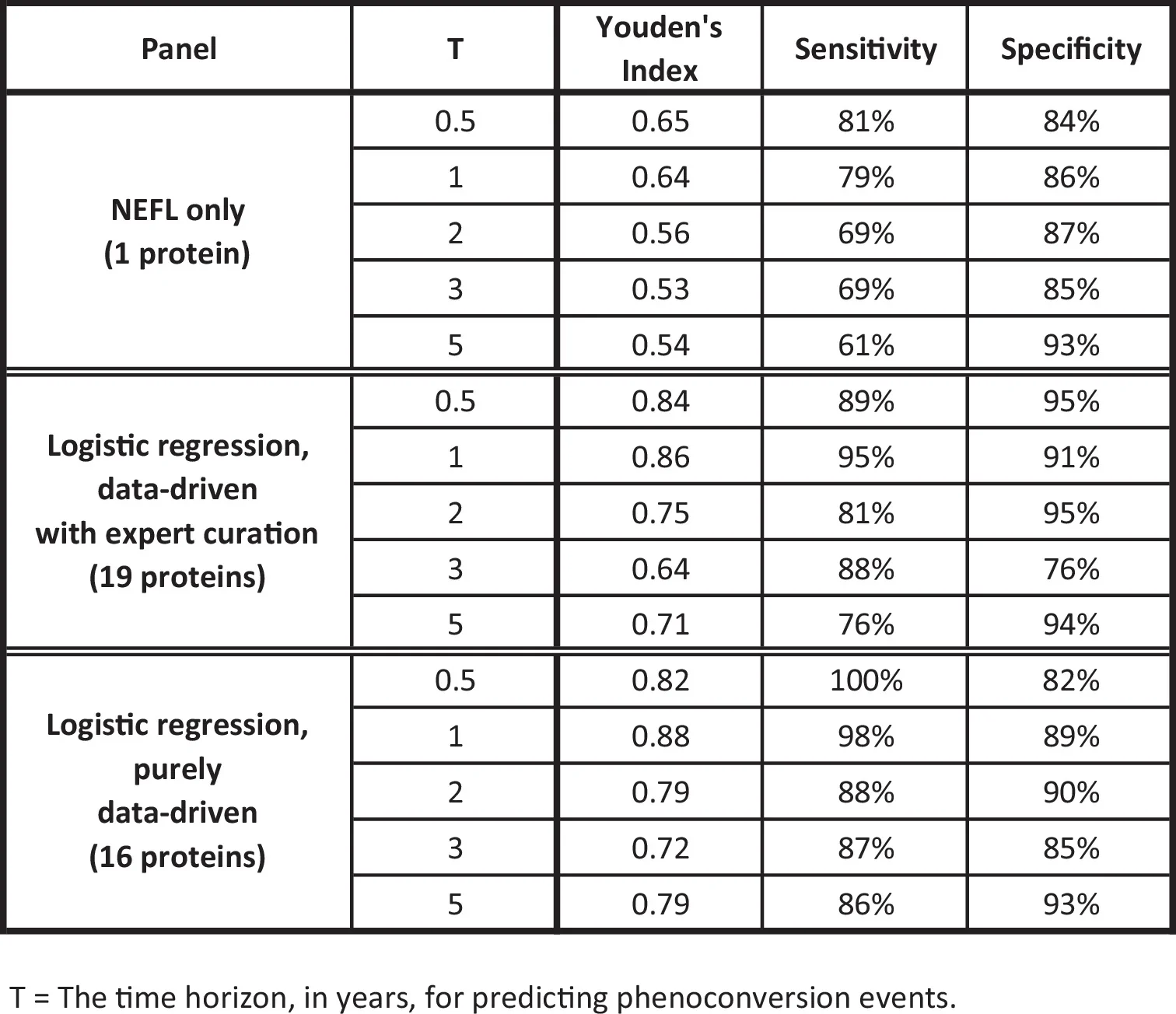
Accuracy of protein panels in phenoconversion event prediction

**Extended Data Table 5:**
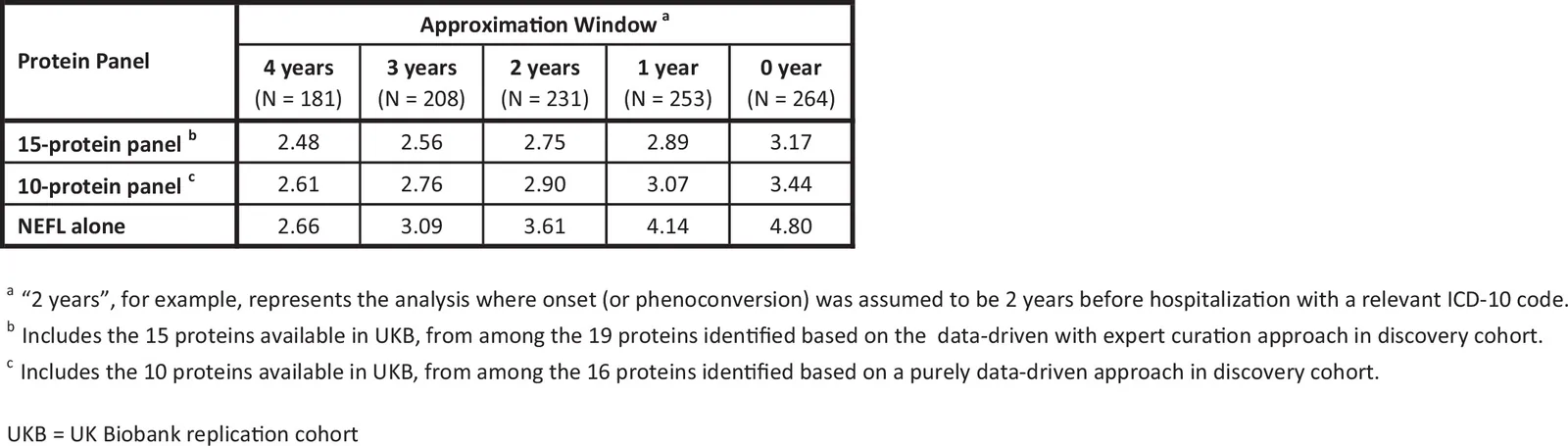
Sensitivity analysis: MAE values for protein prediction panels in replication cohort, across a range of approximation windows

## Data Availability

Access to discovery cohort Olink proteomic data (and corresponding phenotypic data) is controlled to protect study blinding and pathogenic variant carrier confidentiality. Requests for access to this dataset should be directed to the corresponding author (mbenatar@med.miami.edu). Data requests will be reviewed based on compliance with the informed consent and IRB approvals under which the original samples and phenotypic data have been collected, scientific merit and feasibility, appropriateness of the investigator’s qualifications and resources to protect the data. The University of Miami may then release said data via a Data Transfer Agreement. Access to Olink data from the UKB can be obtained through controlled access via their web portal (www.ukbiobank.ac.uk).

## Code availability

Codes used for analyses in this study are available on https://github.com/ranxm2/ALS_protein_longitudinal.
