# Supplementary material for: Longitudinal plasma proteomics predict phenoconversion to clinically manifest ALS
Source: Nat Med. 2026 Jul 27;32(9):3417–29. doi: 10.1038/s41591-026-04528-x (PMC13577924; doi:10.1038/s41591-026-04528-x)
Supplement: Supplementary file 1 [file 41591_2026_4528_MOESM1_ESM.pdf]

nature medicine

Supplementary information https://doi.org/10.1038/s41591-026-04528-x

Longitudinal plasma proteomics predict phenoconversion to clinically manifest ALS

In the format provided by the authors and unedited

Supplementary Figure S1. Protein biomarker trajectories: phenoconverters
(A) heatmap and **(B)** trajectories of 73 protein biomarkers that changed pre-symptomatically, using pre- and post-conversion data from n=33 phenoconverters. **(C)** heatmap and **(D)** trajectories of 52 protein biomarkers that changed pre-symptomatically, using only pre-conversion data from n=33 phenoconverters. Heatmaps, derived from generalized additive mixed model (GAMM) analyses, depict proteins with significant changes in their relative abundance, as compared to sex-age matched controls by first layer GAMM, during the pre-symptomatic stage of disease. The color scale reflects the direction and magnitude of change, with warmer colors (red) indicating increased concentrations and cooler colors (blue) indicating decreased concentration relative to controls. The number in each cell represents the average relative protein abundance by first layer GAMM, during the indicated time interval before phenoconversion. Empty cells denote time intervals where differences, as compared to controls, were not statistically significant. Proteins are grouped by functional modules (see Fig. 1D), as indicated by the color bar in the first column. In trajectory plots, also derived from GAMM analyses, the Y-axis denotes the log2 fold change in protein relative to age- and sex-matched healthy controls by first layer GAMM. The dots and connecting lines show the longitudinal data from individual participants. The black curve depicts the estimated mean longitudinal trajectory of protein abundance; the shaded band denotes the 95% Bayesian credible interval around the fitted mean trend. Horizontal red and blue lines indicate the time periods during which protein levels of phenoconverters were, respectively, significantly higher (log2FC>0) or lower (log2FC<0) compared to matched controls by first layer GAMM. The numeric labels mark the approximate time at which the significant deviation first appeared.

Supplementary Fig. S1A

Protein biomarker trajectories: phenoconverters (pre- and post-conversion data)

Module	-Y16	-Y15	-Y14	-Y13	-Y12	-Y11	-Y10	-Y9	-Y8	-Y7	-Y6	-Y5	-Y4	-Y3	-Y2	-Y1	Y1	Y2	Y3	Y4	Protein
Skeletal Muscle	-0.98	-0.89	-0.74	-0.61											0.33	0.54	0.93	1.34	1.75	1.97	MYL11
Skeletal Muscle	-1.57	-1.50	-1.36	-1.20	-1.01	-0.81							0.51	0.54	0.64	0.87	1.25	1.73	2.22	2.47	MYH1
Skeletal Muscle	-0.50	-0.47	-0.42	-0.36	-0.30	-0.27							0.21	0.28	0.37	0.48	0.61	0.75	0.90	0.98	DTNB
Skeletal Muscle	-0.71	-0.66	-0.58	-0.50	-0.41	-0.35								0.28	0.36	0.50	0.70	0.93	1.18	1.31	SYNM
Skeletal Muscle	-1.53	-1.43	-1.25	-1.06	-0.84	-0.67									0.43	0.68	1.14	1.61	2.08	2.33	MYBPC2
Skeletal Muscle	-1.11	-1.07	-0.98	-0.90	-0.79	-0.65										0.52	0.96	1.42	1.83	2.03	MYOM3
Skeletal Muscle	-0.52	-0.51	-0.48	-0.46	-0.42	-0.38	-0.33	-0.28							0.24	0.36	0.48	0.62	0.75	0.82	NOS1
Skeletal Muscle	-0.88	-0.84	-0.77	-0.69	-0.62	-0.55	-0.48	-0.41	-0.33						0.28	0.38	0.52	0.67	0.83	0.91	TTN
Skeletal Muscle	-0.99	-0.96	-0.90	-0.83	-0.77	-0.70	-0.62	-0.53	-0.44	-0.35				0.27	0.35	0.49	0.64	0.79	0.95	1.03	DMD
Skeletal Muscle	-1.85	-1.77	-1.61	-1.46	-1.31	-1.15	-1.00	-0.85	-0.69	-0.54	-0.45					0.43	0.54	0.70	0.85	0.93	SMTN
Skeletal Muscle		-1.12	-0.96												0.58	0.94	1.57	2.24	2.92	3.27	CSRP3
Skeletal Muscle		-0.76	-0.78	-0.79	-0.74	-0.62									0.43	0.79	1.37	1.96	2.49	2.76	MYBPC1
Skeletal Muscle		-0.73	-0.70	-0.65	-0.61	-0.57	-0.52	-0.47	-0.41	-0.35						0.24	0.42	0.73	1.04	1.21	MYL1
Skeletal Muscle			-0.36	-0.35	-0.33	-0.32											0.30	0.43	0.52	0.55	CAPN3
Skeletal Muscle			-0.35	-0.37	-0.38	-0.37	-0.34	-0.30	-0.24	-0.19				-0.15	-0.17	-0.23	-0.31	-0.41	-0.51	-0.56	ART3
Skeletal Muscle											0.35	0.33			0.28	0.47	0.88	1.36	1.86	2.12	ANKRD2
Skeletal Muscle													0.30	0.33	0.45	0.70	1.04	1.39	1.74	1.92	CA3
Skeletal Muscle														0.48	0.66	1.05	1.60	2.21	2.82	3.14	TNNC1
Skeletal Muscle														0.39	0.51	0.80	1.19	1.61	2.05	2.28	CMYA5
Skeletal Muscle														0.21	0.27	0.40	0.55	0.72	0.89	0.98	KLHL41
Skeletal Muscle															0.45	0.72	1.32	2.00	2.67	3.00	MYBPH
Skeletal Muscle															0.37	0.60	1.02	1.47	1.92	2.15	ACTN2
Skeletal Muscle															0.40	0.64	0.98	1.34	1.72	1.91	MYL3
Skeletal Muscle															0.43	0.64	0.91	1.19	1.47	1.61	MYOM2
Skeletal Muscle															0.24	0.41	0.70	1.01	1.32	1.48	NEB
TNF-Mediated Signaling	-0.71	-0.66	-0.58	-0.52									0.25	0.32	0.40	0.48	0.56	0.64	0.72	0.76	TAX1BP1
TNF-Mediated Signaling	-1.01	-0.97	-0.88	-0.79	-0.70	-0.61	-0.52	-0.43	-0.34	-0.27					0.22	0.29	0.38	0.47	0.56	0.60	NFAT5
TNF-Mediated Signaling	-1.49	-1.42	-1.30	-1.17	-1.05	-0.92	-0.80	-0.67	-0.55	-0.45						0.37	0.46	0.59	0.71	0.77	EIF2AK3
TNF-Mediated Signaling	-1.93	-1.84	-1.70	-1.55	-1.40	-1.25	-1.10	-0.96	-0.81	-0.66	-0.51						0.45	0.53	0.68	0.76	MAP3K5
TNF-Mediated Signaling									0.24	0.28	0.30	0.31	0.31	0.34	0.43	0.59	0.79	1.01	1.26	1.39	EDA2R
TNF-Mediated Signaling											0.25	0.26	0.26	0.26	0.30	0.39	0.56	0.76	0.94	1.04	TNFRSF12A
Regeneration													0.19	0.21	0.30	0.46	0.70	0.97	1.25	1.39	RBFOX3
Regeneration															0.66	1.56	2.50	3.02	3.33	3.48	NEFL
ECM & ECM Interaction									0.17	0.19	0.22	0.25	0.27	0.30	0.32	0.35	0.38	0.40	0.43	0.44	COL6A3
Others	-0.75	-0.73											0.28	0.36	0.45	0.54	0.63	0.71	0.80	0.85	SERPINB8
Others	-0.40	-0.38												0.19	0.22	0.27	0.32	0.36	0.41	0.43	THBS4
Others	-0.48	-0.47													0.16	0.19	0.24	0.28	0.33	0.35	EZR
Others	-0.93	-0.91															0.44	0.52	0.61	0.66	SDCCAG8
Others	-0.65	-0.62	-0.56	-0.50	-0.44	-0.38	-0.33	-0.27								0.20	0.25	0.31	0.37	0.40	MPHOSPH9
Others	-1.30	-1.24	-1.12	-1.01	-0.89	-0.78	-0.66	-0.55	-0.43	-0.33					0.27	0.38	0.49	0.61	0.72	0.78	STX4
Others	-1.35	-1.28	-1.17	-1.05	-0.93	-0.82	-0.70	-0.58	-0.47	-0.35	-0.28				0.26	0.35	0.47	0.59	0.70	0.76	STRN
Others	-1.38	-1.32	-1.20	-1.08	-0.96	-0.84	-0.71	-0.59	-0.52							0.41	0.50	0.62	0.74	0.80	KIFC3
Others	-1.86	-1.76	-1.59	-1.41	-1.23	-1.06	-0.94										0.67	0.78	0.90	0.97	JCAD
Others		-0.49	-0.48	-0.46	-0.44	-0.41	-0.38	-0.33	-0.28	-0.25	-0.22	-0.21	-0.20	-0.16			0.18	0.29	0.43	0.51	HPCAL4
Others			-0.46	-0.44											0.37	0.63	1.01	1.42	1.84	2.05	CORO6
Others			-1.06	-1.13	-1.20	-1.24	-1.24	-1.18	-1.06	-0.85	-0.61				0.42	0.54	0.67	0.83	1.01	1.10	IRAG2
Others					-0.49	-0.48	-0.45	-0.41	-0.37						0.30	0.41	0.54	0.67	0.81	0.88	TRAK1
Others								0.21	0.25	0.28	0.28	0.25	0.21	0.19	0.23	0.33	0.52	0.77	1.06	1.22	AFAP1L1
Others								0.19	0.20	0.22	0.22	0.21	0.19	0.18	0.21	0.28	0.39	0.54	0.69	0.77	SORBS1
Others									0.33	0.38	0.43	0.48	0.53	0.58	0.63	0.69	0.74	0.79	0.84	0.86	CAP2
Others									0.13	0.16	0.18	0.18	0.18	0.19	0.22	0.27	0.33	0.40	0.46	0.50	PHOSPHO1
Others									0.19	0.20	0.21	0.20	0.17					-0.21	-0.28	-0.33	ITGB6
Others										0.32	0.38	0.44	0.50	0.56	0.62	0.68	0.74	0.80	0.86	0.89	PTGES2
Others										0.27	0.33	0.36	0.33	0.28	0.25	0.34	0.59	0.90	1.20	1.35	FABP3
Others										0.20	0.19	0.18		0.18	0.25	0.39	0.57	0.78	0.99	1.10	HSPB6
Others										0.16	0.16	0.15	0.14								CRTAC1
Others											0.30	0.37	0.43	0.50	0.56	0.62	0.68	0.74	0.80	0.83	NGRN
Others												0.29	0.35	0.44	0.56	0.70	0.86	1.03	1.21	1.31	CALCA
Others												0.24	0.28	0.33	0.40	0.47	0.55	0.62	0.70	0.74	PRND
Others												0.22	0.25	0.32	0.39	0.46	0.53	0.59	0.66	0.70	OTUD4
Others													0.28	0.35	0.43	0.53	0.65	0.77	0.89	0.95	USP28
Others													0.29	0.36	0.43	0.51	0.59	0.67	0.74	0.78	MYH9
Others														0.36	0.44	0.64	0.90	1.19	1.49	1.64	DUSP29
Others														0.38	0.46	0.55	0.64	0.73	0.83	0.87	FYCO1
Others														0.23	0.31	0.41	0.53	0.67	0.81	0.89	ADAM12
Others														0.35	0.40	0.46	0.53	0.59	0.65	0.69	WASF3
Others															0.23	0.36	0.54	0.74	0.95	1.05	HS6ST2
Others															0.35	0.40	0.52	0.66	0.82	0.90	KLK4
Others															0.37	0.43	0.51	0.60	0.68	0.73	CALCOCO1
Others															0.14	0.23	0.40	0.59	0.79	0.88	TXLNB
Others																0.28	0.45	0.68	0.96	1.12	CALCB
Others																0.26	0.45	0.69	0.92	1.04	MEGF10
Others																0.17	0.27	0.51	0.80	0.95	CD276

Phenoconversion

Module
- Skeletal Muscle
- TNF-Mediated Signaling
- Regeneration (incl. Neurofilament)
- ECM & ECM Interaction
- Others

Log2FC
Increase
4
2
0
-2
-4
Decrease

Supplementary Fig. S1B

Protein biomarker trajectories: phenoconverters
(pre- and post-conversion data)

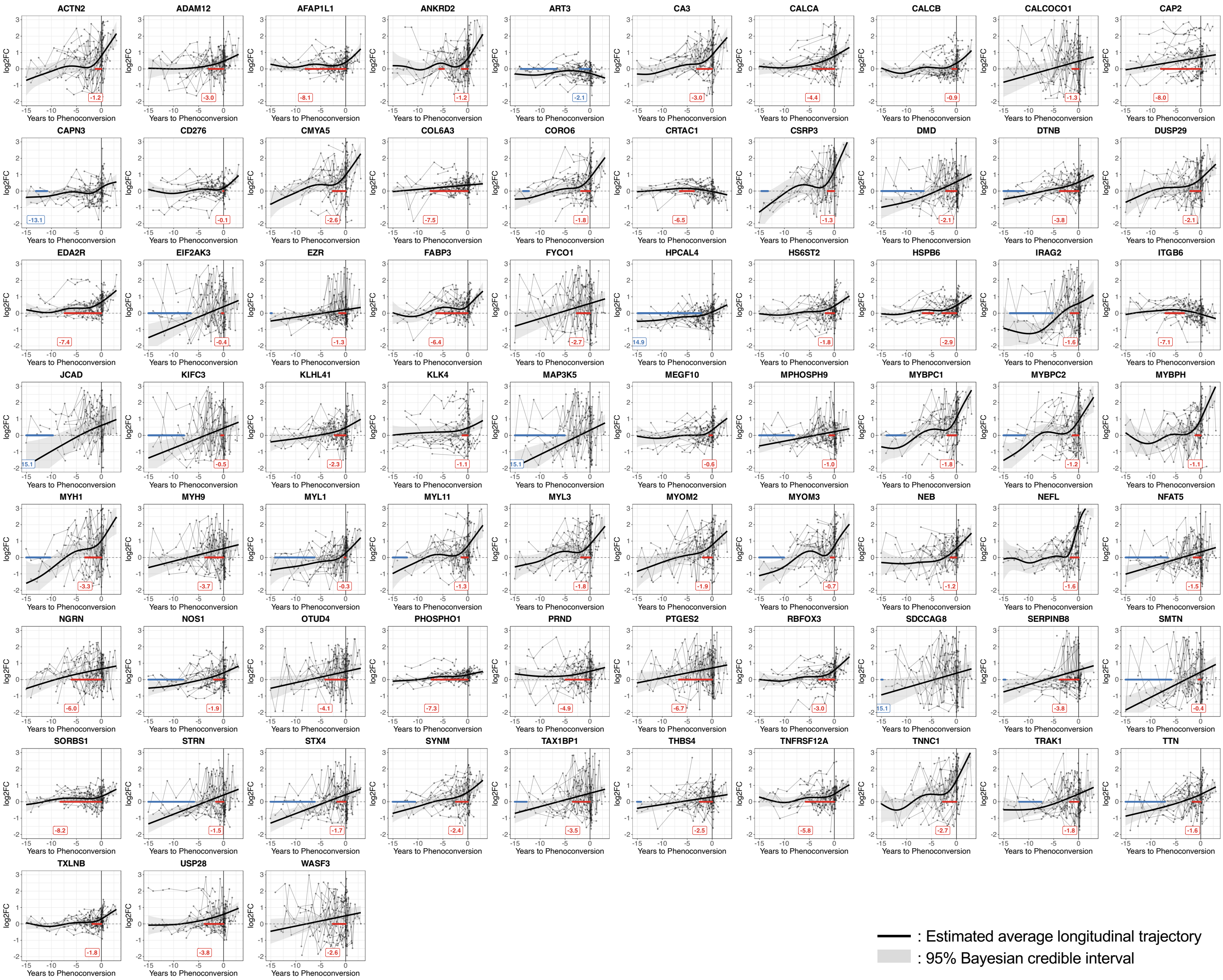

Supplementary Fig. S1C

Protein biomarker trajectories: phenoconverters (pre-conversion data only)

Module	Protein	-Y16	-Y15	-Y14	-Y13	-Y12	-Y11	-Y10	-Y9	-Y8	-Y7	-Y6	-Y5	-Y4	-Y3	-Y2	-Y1
Skeletal Muscle	CMYA5	-0.71	-0.63	-0.54								0.35	0.38	0.43	0.48	0.54	0.61
Skeletal Muscle	DTNB	-0.43	-0.40	-0.34	-0.28							0.18	0.21	0.27	0.33	0.39	0.45
Skeletal Muscle	SYNM	-0.66	-0.62	-0.54	-0.46	-0.39	-0.33							0.25	0.30	0.37	0.45
Skeletal Muscle	NOS1	-0.61	-0.57	-0.51	-0.45	-0.39	-0.32	-0.27							0.20	0.24	0.30
Skeletal Muscle	MYBPC1	-0.91	-0.85	-0.74	-0.64	-0.53	-0.47								0.37	0.45	0.53
Skeletal Muscle	CSRP3	-1.16	-1.04	-0.83	-0.70										0.41	0.46	0.58
Skeletal Muscle	MYH1	-1.62	-1.52	-1.33	-1.14	-0.93	-0.71						0.50	0.54	0.54	0.50	0.46
Skeletal Muscle	TTN	-0.84	-0.80	-0.72	-0.64	-0.57	-0.49	-0.42	-0.34	-0.29							0.28
Skeletal Muscle	MYL1	-0.82	-0.79	-0.73	-0.68	-0.63	-0.57	-0.52	-0.47	-0.41	-0.36	-0.30	-0.25	-0.22			
Skeletal Muscle	MYL11	-0.76	-0.70	-0.57	-0.48												
Skeletal Muscle	SMTN	-1.33	-1.29	-1.22	-1.15	-1.08	-1.00	-0.90	-0.79	-0.66	-0.55					0.49	0.67
Skeletal Muscle	MYBPC2	-1.43	-1.32	-1.13	-0.93	-0.72	-0.60										0.46
Skeletal Muscle	MYOM3	-1.13	-1.08	-0.99	-0.91	-0.82	-0.68	-0.56									
Skeletal Muscle	DMD		-0.66	-0.65	-0.63	-0.61	-0.57	-0.53	-0.46	-0.40					0.34	0.47	0.63
Skeletal Muscle	MYL3											0.40	0.40	0.36		0.35	0.47
Skeletal Muscle	CA3											0.26	0.30	0.35	0.41	0.47	0.53
Skeletal Muscle	TNNC1												0.43	0.50	0.58	0.67	0.76
Skeletal Muscle	KLHL41														0.18	0.22	0.26
Skeletal Muscle	MYOM2																0.42
Skeletal Muscle	ACTN2																0.35
TNF-Mediated Signaling	NFAT5	-0.80	-0.76	-0.70	-0.64	-0.58	-0.51	-0.45	-0.37	-0.31					0.23	0.31	0.42
TNF-Mediated Signaling	MAP3K5	-1.43	-1.40	-1.35	-1.29	-1.23	-1.16	-1.08	-0.97	-0.83	-0.67	-0.56					0.58
TNF-Mediated Signaling	EIF2AK3			-0.87	-0.85	-0.83	-0.81	-0.77	-0.72	-0.63	-0.53				0.38	0.50	0.74
TNF-Mediated Signaling	EDA2R									0.24	0.28	0.31	0.33	0.34	0.36	0.42	0.49
TNF-Mediated Signaling	TAX1BP1												0.27	0.34	0.46	0.58	0.70
Regeneration (incl. Neurofilament)	RBFOX3												0.20	0.23	0.27	0.32	0.37
Regeneration (incl. Neurofilament)	NEFL															0.49	1.19
Others	NGRN	-0.94	-0.82	-0.65								0.32	0.32	0.36	0.45	0.59	0.77
Others	SERPINB8	-0.88	-0.81	-0.70	-0.59							0.25	0.32	0.44	0.55	0.67	0.78
Others	CORO6	-0.52	-0.48	-0.42	-0.35									0.26	0.31	0.37	0.44
Others	EZR	-0.55	-0.52	-0.46	-0.40	-0.36									0.18	0.24	0.29
Others	HPCAL4	-0.51	-0.49	-0.46	-0.43	-0.40	-0.37	-0.35	-0.32	-0.29	-0.26	-0.23	-0.20	-0.17	-0.15		
Others	STX4	-0.91	-0.88	-0.82	-0.76	-0.70	-0.64	-0.57	-0.49	-0.39	-0.32				0.27	0.37	0.52
Others	STRN	-0.95	-0.93	-0.88	-0.84	-0.79	-0.73	-0.66	-0.57	-0.47	-0.36				0.27	0.37	0.52
Others	JCAD	-2.01	-1.90	-1.69	-1.49	-1.29	-1.08	-0.97								0.76	0.97
Others	IRAG2			-0.86	-0.91	-0.99	-1.05	-1.07	-1.04	-0.93	-0.75	-0.56			0.45	0.63	0.92
Others	PHOSPHO1									0.11	0.13	0.15	0.17	0.19	0.22	0.24	0.26
Others	CAP2										0.35	0.39	0.44	0.49	0.53	0.58	0.62
Others	CALCA											0.28	0.32	0.37	0.43	0.48	0.54
Others	HSPB6											0.16	0.19	0.21	0.24	0.27	0.30
Others	MYH9												0.32	0.38	0.47	0.56	0.65
Others	USP28												0.27	0.31	0.36	0.42	0.47
Others	FYCO1													0.46	0.60	0.75	0.90
Others	CALCOCO1													0.40	0.48	0.59	0.70
Others	DUSP29													0.31	0.36	0.42	0.48
Others	HS6ST2													0.17	0.19	0.22	0.24
Others	TXLNB													0.11	0.13	0.15	0.17
Others	KIFC3														0.40	0.49	0.65
Others	TRAK1														0.29	0.37	0.51
Others	PDZD2														0.28	0.36	0.51
Others	MPHOSPH9														0.19	0.24	0.33
Others	CALCB																0.26

Phenoconversion (after -Y1)

Module
- Skeletal Muscle
- TNF-Mediated Signaling
- Regeneration (incl. Neurofilament)
- ECM & ECM Interaction
- Others

Log2FC
Increase
4
2
0
-2
-4
Decrease

Supplementary Fig. S1D

Protein biomarker trajectories: phenoconverters (pre-conversion data only)

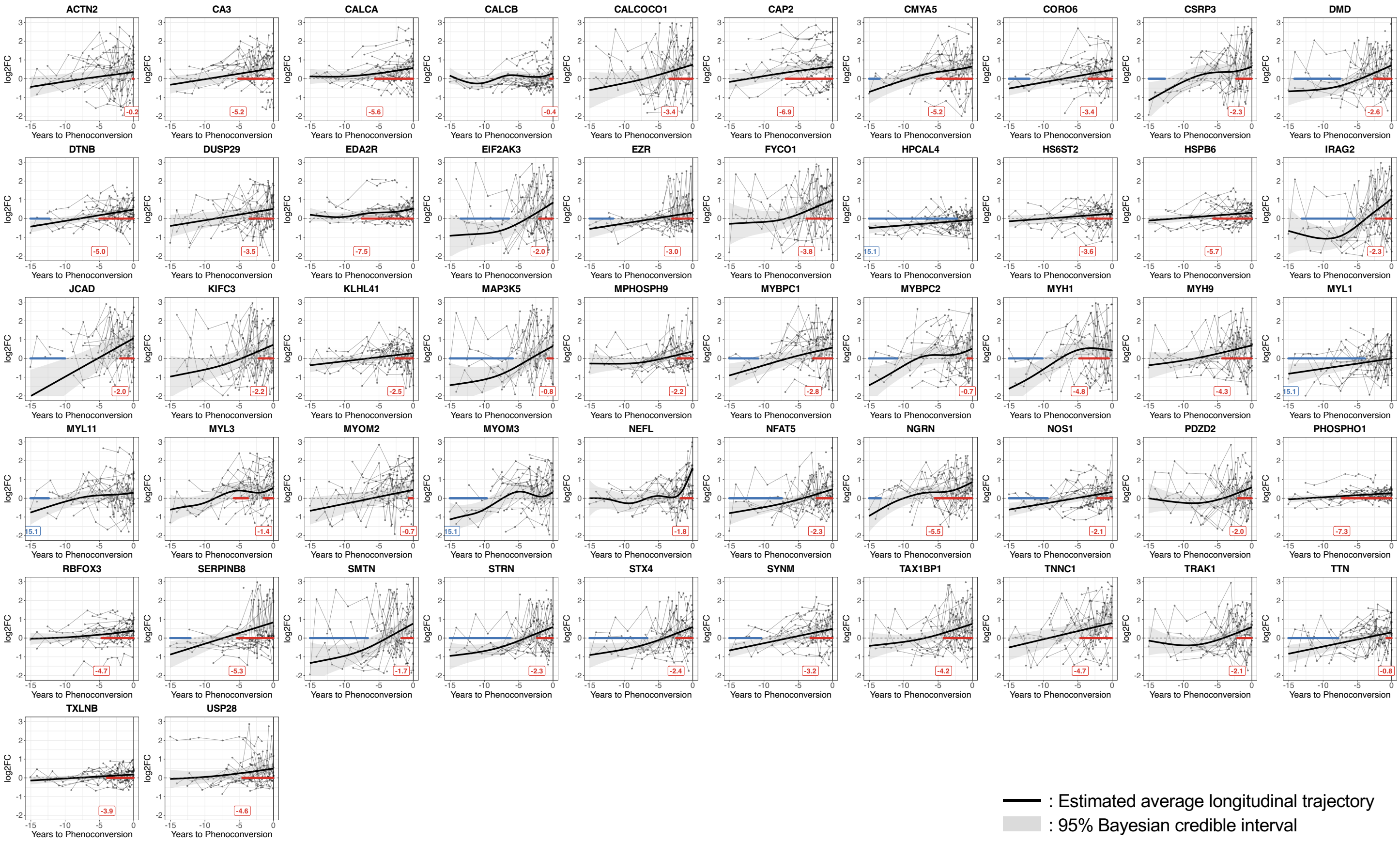

Supplementary Figure S2. Comparison of phenoconversion event prediction model performance, across different timeframes and machine learning algorithms

Heatmap showing the optimal 5-fold cross-validation AUCs for phenoconversion event prediction among n=33 phenoconverters and n=10 pre-symptomatic carriers. The five columns correspond to five prediction timeframes (T = 0.5, 1, 2, 3, and 5 years), and the seven rows correspond to the seven machine learning approaches evaluated: five algorithms (logistic regression, support vector machine, boosting, random forest, and XGBoost) using forward greedy search for feature selection, along with two additional logistic regression models employing Elastic Net and LASSO regularization. The 137 proteins differentially regulated in ALS vs. controls were included in these analyses. SVM = support vector machine; XGBoost = Extreme Gradient Boosting; LASSO = Least Absolute Shrinkage and Selection Operator.

Supplementary Fig. S2

Comparison of phenoconversion event prediction model performance, across different timeframes and machine learning algorithms

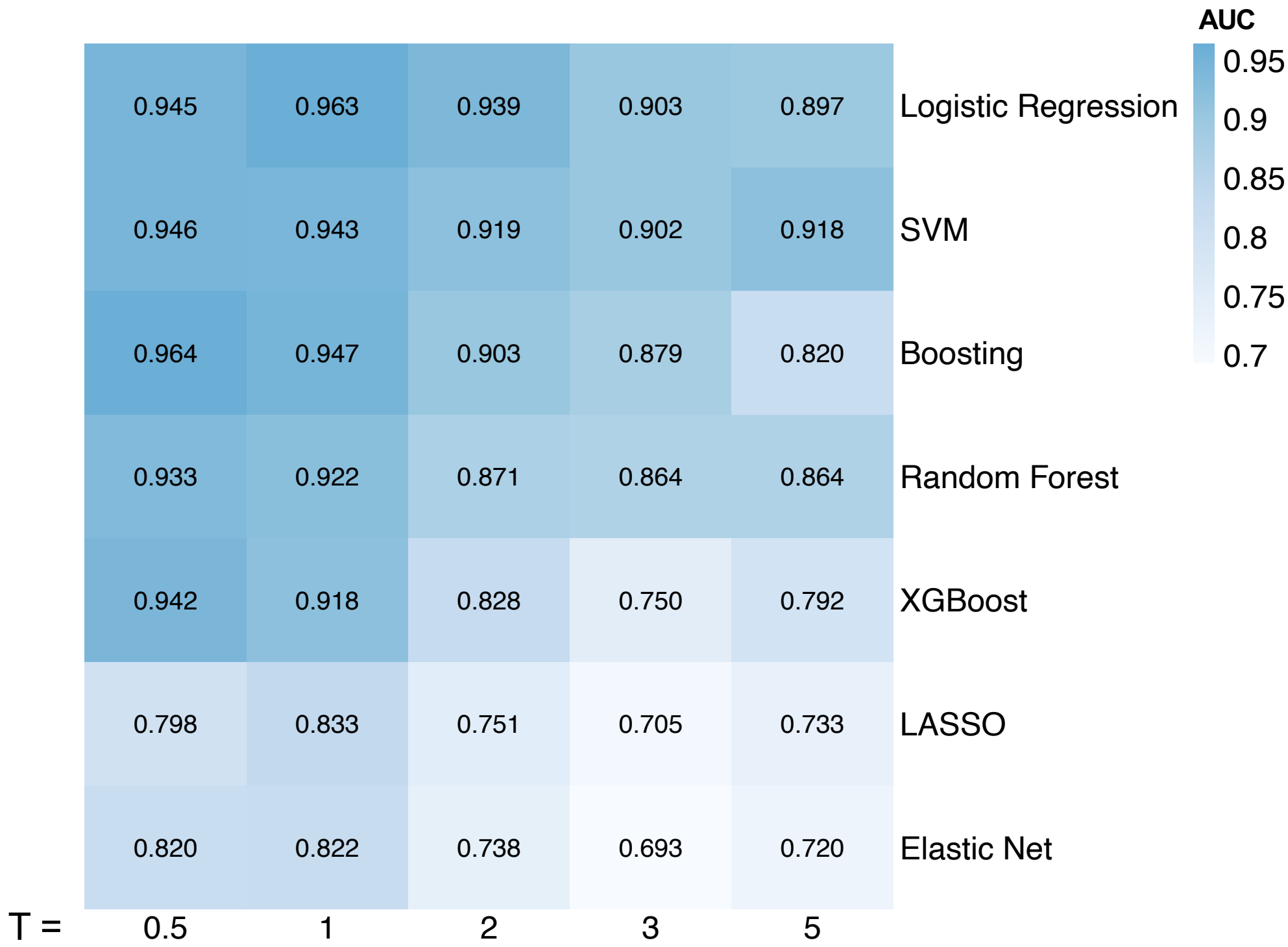

SUPPLEMENTARY INFORMATION – DETAILED METHODS

(A) ***Parent studies: cohort descriptions, ethics approvals, sample collection and processing***

This proteomic study included participants from 3 parent studies: *Pre-fALS*, the Clinical Research in ALS (CRiALS) Biomarker study, and the CReATe Consortium Phenotype-Genotype-Biomarker (PGB1) study. Details of *Pre-fALS* (NCT00317616) have previously been described [1-5]. Briefly, *Pre-fALS* is a single-center study that recruits, from across North America, individuals who are carriers of any ALS (or ALS/FTD) associated pathogenic variant and who, at the time of enrollment, are clinically pre-symptomatic for ALS and FTD. Since its inception in 2007, *Pre-fALS* has been broadly inclusive regardless of participants' geolocation and genotype, to maximize the representativeness of the study cohort and generalizability of the results. Participants undergo screening [1] as well as genetic testing and counseling [2] as needed. Eligible participants are enrolled in the *Pre-fALS* Cohort and followed longitudinally, with detailed phenotypic evaluations, biospecimen collections, and multi-modal biomarker procedures. If evidence of clinically manifest disease is found during baseline evaluation, the individual is excluded from the *Pre-fALS* Cohort and enrolled, instead, in the CRiALS Biomarker study (see below) and referred for clinical care. Notably, we do not regard these individuals as phenoconverters, a designation reserved for those who were pre-symptomatic at baseline and subsequently developed clinically manifest ALS or FTD. Among Cohort enrollees, the subset who choose not to learn their genetic test results ("non-disclosure" group) [1] comprise those who do and do not carry a pathogenic variant; the latter subgroup are effectively controls. CRiALS (NCT00136500) Biomarker is a companion study to *Pre-fALS*, serving to recruit both healthy controls and patients with clinically manifest ALS, to aid the interpretation of pre-symptomatic data from *Pre-fALS*. CRiALS Biomarker study procedures and clinical assessments mirror those used in *Pre-fALS*. Additional participants with clinically manifest ALS were drawn from CReATe PGB1 (NCT02327845), a multi-center natural history study of individuals with ALS and related disorders, in which participants were evaluated serially to acquire longitudinal phenotypic data and biospecimens. PGB1 participants included in this report were all from the University of Miami site.

All 3 studies were approved by the University of Miami institutional review board (IRB), which also serves as the single IRB of record for the CReATe Consortium, and all study participants provided written informed consent. The University of Miami IRB operates under the umbrella of the University of Miami Human Subjects Research Office (FWA00002247).

Plasma samples were collected, processed, and stored according to strict standard operating procedures. Briefly, blood was collected in K2 EDTA tubes, centrifuged at 1,750 x g for 10 minutes at 4°C, and aliquoted for storage at −80°C.

(B) ***Statistical analysis***

We applied machine learning methods to predict the occurrence and timing of phenoconversion. Our method for discovery progressed through five steps: differentially expressed protein selection, temporal dynamics protein modeling, time-to-phenoconversion Cox modeling, phenoconversion event prediction, and phenoconversion timing estimation. All statistical tests were two-sided, and all analyses were performed in R 4.4.0.

Step 1. Differentially expressed proteins selection

Step 1a. Differentially regulated proteins identification

The goal in this step was to select a subset of proteins differentially expressed between clinically manifest ALS and healthy controls (i.e. disease state biomarkers) from the ~5,300 in the Olink Explore HT panel for downstream analysis. This step was based on the rationale that proteins

differentially expressed between clinically manifest ALS patients and healthy controls are likely ALS-related and would be strong candidates to predict phenoconversion among pathogenic variant carriers. To ensure no overlap between samples used in this step and those used to build prediction models, we included in this step only study participants from the clinically manifest (N = 35) and healthy control (N = 59) groups, each with Olink data from 126 visits (Table 1). Since all but 21 controls contributed multiple samples, we employed a mixed-effects model with a random intercept to account for within-person correlation. We also included age at sample collection, sex, and genotype group (*SOD1* A4V, *SOD1* non-A4V, *C9orf72*, other pathogenic variants) as covariates. The Benjamini-Hochberg (BH) procedure was applied to control the false discovery rate (FDR) [6] at a significance threshold of 0.05.

Step 1b. Biological implications

Pathway enrichment analysis was performed using the 'gost' function in the gprofiler2 package (version 0.2.3), which provides functional profiling by mapping differentially expressed proteins to Gene Ontology (GO), Kyoto Encyclopedia of Genes and Genomes (KEGG), Reactome, and other databases [7]. Statistical significance was defined as an FDR-adjusted P value < 0.05.

Protein-protein interaction (PPI) networks were constructed to investigate the functional connectivity among the differentially expressed proteins. Interactions were queried using the STRING database (version 11.5), which integrates evidence from experimental data, computational prediction, co-expression, curated databases, and literature mining [8]. To identify biologically coherent subnetworks, we applied Markov Cluster Algorithm (MCL) clustering using STRING's built-in implementation, with an inflation parameter set to 3 to control cluster granularity.

Step 2. Temporal dynamics protein modeling

To evaluate whether the differential abundance observed between clinically manifest ALS patients and healthy controls can also be detected prior to phenoconversion, we modeled the longitudinal trajectories of the subset of proteins identified in Step 1, using a two-layer generalized additive mixed model (GAMM) framework [9].

In the first layer, we modeled the protein trajectory in healthy controls only and included sex as a fixed covariate, a random intercept to account for within-subject correlation, and a smooth function of age ($\sum_{k=1}^{10} f_k^{(p)}(\text{Age}_{ij})$) to capture non-linear age-related changes in protein abundance:

$$\text{Y}_{ij}^{(p)} = \beta_0^{(p)} + \sum_{k=1}^{10} f_k^{(p)}(\text{Age}_{ij}) + \beta_2^{(p)}\text{Sex}_i + b_i^{(p)} + \epsilon_{ij}^{(p)},$$

$$where\ b_{ij}^{(p)} \sim N(0, \tau_i^2), \qquad \epsilon_{ij}^{(p)} \sim N(0, \tau^2),$$

$\text{Y}_{ij}^{(p)}$ is the observed normalized abundance of protein p for individual i at visit j, k is the number of component functions (range: 1-10), b is the individual random effect, and ϵ is the random error.

In the second layer, we modeled the relative protein abundance (δ) – defined as the deviation from the expected protein abundance in an age- and sex-matched healthy control based on model in the first layer – over time. The GAMM model in the second layer included a random intercept for individual-level variation and a smooth function ($\sum_{k=1}^{10} g_k^{(p)}(\text{t}_{ij})$) of time to phenoconversion (t_{ij}). This allowed us to characterize non-linear protein trajectory patterns as each individual approached phenoconversion.

$$\delta_{ij}^{(p)} = \gamma_0^{(p)} + \sum_{k=1}^{10} g_k^{(p)}(\text{t}_{ij}) + c_i^{(p)} + \vartheta_{ij}^{(p)},$$

$$where\ c_{ij}^{(p)} \sim N(0, \varphi_i^2), \qquad \vartheta_{ij}^{(p)} \sim N(0, \varphi^2).$$

To assess the statistical significance of the disease effect in each model, we applied the BH procedure on smooth term to control the false discovery rate at a significance threshold of 0.05.

For each protein that exhibited a significant pre-symptomatic change, we determined the earliest time of this change by first constructing the pointwise 95% Bayesian credible interval (CrI) around the fitted curve of the longitudinal protein trajectory, with standard errors obtained from the Bayesian posterior covariance matrix of the spline coefficients under REML estimation in the mgcv package [9]; these intervals have good frequentist across-the-function coverage properties [10]. We then identified the time period(s) during which this CrI excluded 0, indicating a significant difference from age- and sex-match controls, and considered the start of this period to be the earliest time of pre-symptomatic change. If for a protein more than one time period were identified, we retained the one closest to phenoconversion in downstream analyses, given the higher density of data in this period (see ART3 in Supplementary Fig. S2 as an example). All significant pre-symptomatic time periods are nonetheless shown in Supplementary Fig. S2 and S3 for reference.

In primary analyses, the models included data from both phenoconverters and those with clinically manifest ALS. For the latter group, reported onset of weakness was used as a proxy for phenoconversion. Secondarily, we repeated these models but included only data from phenoconverters (pre- and post-conversion) or only pre-conversion visits from phenoconverters.

Step 3. Time-to-phenoconversion Cox regression

To assess the association between protein biomarker and subsequent phenoconversion, we performed time-to-event analyses using Cox proportional hazards models and baseline protein biomarker data from phenoconverters (i.e. those who met phenoconversion endpoint) and pre-symptomatic individuals (i.e. those who were censored). We first fitted univariate Cox models, including relative protein abundance as the predictor and adjusting for covariates (age, sex, and genotype group). Multiple testing was controlled for using the BH procedure (FDR < 0.05). For multivariable prediction, we constructed partially penalized Cox models with LASSO regularization, penalizing only the protein variables while retaining clinical covariates without penalty. The optimal penalty parameter was selected using leave-one-out cross-validation (LOOCV). The LASSO-derived risk score for each participant was calculated as a weighted sum of the selected proteins and covariates, using the regression coefficients estimated from the optimal penalized Cox model. The performance of different protein panels was evaluated by comparing those with high vs. low risk score (median split) using Kaplan-Meier analysis and log-rank test.

To leverage longitudinal proteomic measurements, we additionally explored univariate and LASSO-penalized time-dependent Cox models, in which protein abundance was treated as a time-varying covariate updated at each visit. Univariate models were evaluated using BH procedure with FDR < 0.05, and LASSO penalization was applied to protein variables only, with clinical covariates retained unpenalized and the optimal penalty parameter selected by LOOCV.

Step 4. Phenoconversion event prediction

Using the differentially expressed proteins (i.e., disease state biomarkers) identified in Step 1, this step aimed to build prediction models for whether a pre-symptomatic pathogenic variant carrier will phenoconvert to ALS within a certain timeframe (T), with T ranging from 0.5 to 5 years. To enhance model robustness, we used absolute protein levels (NPX) rather than relative protein abundance (δ) in these prediction models. This choice eliminates the dependence on a reference control population for calibration and ensures applicability in clinical settings where repeated measurements or a similar control reference may not be available. Training data were drawn from the pre-symptomatic and phenoconverter groups, with each visit annotated as either “case” or “control”, depending on,

respectively, whether or not phenoconversion occurred within the specified time period following the visit. For the pre-symptomatic group, all visits with subsequent follow-up time longer than (or equal to) T were annotated as "controls"; the remaining visits were excluded as we do not know whether the participant might still phenoconvert within the T year interval. For the phenoconverter group, all visits where phenoconversion occurred more than T years after the visit were considered "controls", whereas all visits where phenoconversion occurred within T years of the visit were considered "cases". Each phenoconverter's first post-conversion visit was also included and labeled as a "case," while all other post-conversion visits were excluded.

We evaluated seven machine learning approaches: five algorithms (logistic regression, support vector machine, boosting, random forest, and XGBoost) with forward greedy search for feature selection, along with two additional logistic regression models employing Elastic Net and LASSO. Age, sex and genotype group were included as covariates. We found that logistic regression (LR) models achieved high predictive performance using only the relatively small number of proteins from Step 1; the LR models were also preferred given our modest sample size, and their results being readily interpretable (Supplementary Fig. S4). Model training and testing were performed using five-fold cross-validation. Training and testing sets were stratified to maintain a balance of cases and controls. Additionally, to maintain independence between the training and test sets (i.e. to prevent leakage across folds), we ensured that multiple visits from the same participant were assigned to only one of the five folds.

We tested five different timeframes: T = 0.5, 1, 2, 3 and 5 years. For each timeframe, we first ran univariate LR for each protein individually, with covariates for sex, age (at sample collection) and genotype group. From the results, we identified the proteins that returned the best prediction measured by area under the curve (AUC) of a receiver operating characteristic (ROC) curve. Next, we performed a greedy stepwise LR with forward greedy search. Specifically, we progressively added, one at a time, proteins that maximized the incremental (or minimized the decremental) predictive performance among all remaining proteins. Performance was measured using the average AUC across the five-fold cross validation. The model with the best predictive performance in the testing set was selected as the designated model for that timeframe. We counted how many times each of the differentially expressed proteins from Step 1 are included in the five designated models (one for each timeframe). Among the proteins that appeared at least once in the five models (Extended Data Table 3), we further filtered them based on their ranking, the timeframe(s) in which they appeared, as well as biological rationale to arrive at a small core panel of proteins as susceptibility/risk biomarkers. The resulting protein panel was checked for potential multicollinearity using variance inflation factor (VIF) [11]. Across the five timeframes, median (25th-75th percentile) VIF = 2.5 (2.1-4.2), with only 2 proteins (TNNC1 and MYL3) exhibiting VIF > 10, likely reflecting shared biological pathways among muscle-related proteins, but only at shorter timeframes and it did not materially affect model stability – indicating that multicollinearity was not a concern in the final model. Notably, the greedy forward selection procedure evaluates the incremental predictive contribution of each protein conditional on those already included in the model, thereby providing an interpretable feature-selection framework. Because this procedure directly measures the marginal contribution of each candidate protein to model performance, additional feature-importance analyses (e.g., permutation importance or SHAP values) were not performed.

Similarly and in parallel, we undertook a purely data-driven approach (i.e. without expert curation), using greedy forward selection to identify a small panel of proteins that predicted phenoconversion across the different timeframes, based on their average cross-validation AUCs across these timeframes.

To determine the optimal classification threshold of the resulting core protein panels, we used the Youden index derived from the ROC curve. Specifically, for each model we identified the threshold that maximized Youden's J statistic (sensitivity + specificity − 1), which represents the point that optimally balances sensitivity and specificity. This threshold was then used to predict whether an individual would phenoconvert within T years after visit, classifying each individual as either a converter or non-converter.

To illustrate the association between the baseline risk score of multi-protein panels and phenoconversion-free survival, we employed partially penalized LASSO Cox analysis, as well as Kaplan-Meier analysis and log-rank test, as described in Step 3 above.

Step 5. Phenoconversion timing estimation
Our goal in this step was to build disease progression models (DPM) to estimate pre-symptomatic mutation carriers' proximity to time of phenoconversion given the proteomic profile obtained from the individual's most recent visit and baseline visit, similar to the approach employed in a recent study of familial FTD that utilized a Bayesian DPM [12]. For this Step, we used the final protein panel identified in Step 4. Instead of LR, we used a truncated regression model and included only data from the pre-conversion visits of phenoconverters, the only group in whom time-to-phenoconversion (t_{ij}) for individual i at visit j is known. (Note that t_{ij} is negative for visits before phenoconversion.) To avoid the collinearity between age and time-to-phenoconversion, we used relative protein abundance from Step 2 as input features. Given the longitudinal structure of the proteomics data, with repeated measurements collected from the same individuals over time, we used each participant's baseline visit as internal reference to account for inter-individual variability in protein abundance. To model time-to-phenoconversion as a continuous outcome, we employed truncated regression, with an upper bound at zero, ensuring that all predicted values $\hat{t}_{ij}$ were negative. In addition, we included sex and genotype group as covariates. in the model:

$$t_{ij} = h(\theta_0 + \boldsymbol{\theta}_1 \boldsymbol{\delta}_{i1} + \boldsymbol{\theta}_2 \boldsymbol{\delta}_{ij} + \theta_3 \text{Sex}_i + \boldsymbol{\theta}_4 \text{Genotype}_i + \varepsilon_{ij}),$$

$$\text{where } \varepsilon_{ij} \sim N(0, \sigma^2),\ \ h(y) = \begin{cases} y & y < 0 \\ 0 & y \geq 0 \end{cases},$$

and $\boldsymbol{\delta}_{ij}$ is the relative protein abundance vector for individual i at visit j.

The predicted and observed time-to-phenoconversion were compared and summarized using root mean square error (RMSE), mean absolute error (MAE), and Pearson correlation. The performance of the multi-protein panel model was compared to the NEFL-only model using the likelihood ratio test to determine whether the multi-protein panel provided a significant improvement in phenoconversion timing estimation above and beyond NEFL.

(C) _UK Biobank replication_
For replication analysis, we used data from the UK Biobank (UKB) cohort. UKB is a large-scale prospective study that recruited 503,317 participants aged 40-69 years between 2006 and 2010 across the United Kingdom [13, 14], with Olink Explore 3072 data available through the UKB Pharma Proteomics Project (UKB-PPP) for N=54,219 [15]. Data for the present analysis were accessed under application #847687.

The replication cohort consists of five participant groups (Table 1), four of which are similarly defined as in the discovery cohort: heathy control, pre-symptomatic, phenoconverter, and clinically manifest. A fifth group (pre-hospitalization, see below) was also included in the modeling of protein trajectories. From among the UKB-PPP participants, because we need genotype information in order to determine eligibility for the control vs. pre-symptomatic group as well as to adjust for genotype in

replication analyses (as is done in discovery analyses), we included only the N=52,488 for whom whole genome sequencing data were also available and who had neither withdrawn consent nor been lost to follow-up (data field 190 in UKB), as of time of data access in August 2025 (Extended Data Fig. 5).

Among them, N=327 ALS cases were identified using the International Classification of Diseases (ICD-10) code of G12.2 (motor neuron disease; data field 41270) based on self-report at the time of enrollment, or in subsequent hospitalization or death records [16]. From this group, we included only those whose G12.2 codes were from hospitalization records with exact dates (N=286). Among these, N=22 had baseline blood draw for proteomic analysis after the occurrence of a hospitalization record with G12.2. These N=22 comprised the **Clinically Manifest** group in the replication cohort (Table 1). The remaining N=264 ALS cases, whose baseline blood draw occurred before hospitalization record with G12.2, were considered as possible phenoconverters. However, since hospitalization is typically not an early event in the course of ALS, we estimated the approximate phenoconversion time to be two years prior to the G12.2 hospitalization date. After applying this adjustment, N=33 had baseline visits after the approximated time of phenoconversion; they comprised the **Pre-hospitalization** group (Extended Data Fig. 5). The remaining N=231 comprised the **Phenoconverter** group in the replication cohort, with a median (range) interval between the baseline visit and the estimated time of phenoconversion being 4.13 (0.05-11.88) years.

Among the N=52,161 participants without a G12.2 record, we further excluded those with any nervous system disease (ICD-10 codes G00-G99; N=15,767), yielding N=36,394 neurologically healthy individuals. Among them, N=35,722 had no pathogenic variants (ClinVar [17]; accessed October 10, 2025) in any ALS genes classified as limited, moderate, strong, or definite by the ClinGen ALS Spectrum Disorders Gene Curation Expert Panel [18], or in *HNRNPA1*, *UNC13A*, or *SPTLC1*; they comprised the **Healthy Control** group. The N=38 with either a pathogenic mutation in the SOD1 gene or a *C9orf72* repeat expansion of more than 100 repeats [17, 19] comprised the **Pre-symptomatic** group.

For replication analysis, we applied the differential protein expression approach (as described in Step 1 above) to the UKB-PPP cohort, using covariate-matched healthy controls for individuals in the clinically manifest group, with covariates including sex, age, and genotype group. Of the 137 proteins identified in the discovery cohort, 95 were available in UKB due to differences between the Olink Explore 3072 and Olink Explore HT platforms. We plotted the log2fold changes of the overlapping proteins and assessed the correlation between discovery and replication cohorts via Pearson correlation. To analyze the temporal dynamics of proteins in the replication cohort, we applied two-layer generalized additive models (GAMs) instead of two-layer generalized additive mixed models (GAMMs), as the UKB data are cross-sectional rather than longitudinal. Accordingly, individual-level random effects were excluded from the models. In the first-layer GAM, relative protein abundance was modeled with a covariate sex and a smooth term of age. The second-layer GAM modeled relative protein abundance against a smooth term of proxy for time-to-phenoconversion. We then tested our phenoconversion timing estimation models trained on the discovery cohort to baseline protein expression levels in the replication cohort, with covariates including sex, age, and genotype group.

References

1. Benatar M, Wuu J. Presymptomatic studies in ALS: rationale, challenges, and approach. Neurology 2012;79:1732-1739.
2. Benatar M, Stanislaw C, Reyes E, et al. Presymptomatic ALS genetic counseling and testing: Experience and recommendations. Neurology 2016;86:2295-2302.

3. Benatar M, Wuu J, Andersen PM, Lombardi V, Malaspina A. Neurofilament light: A candidate biomarker of pre-symptomatic ALS and phenoconversion. Annals of neurology 2018;84:130-139.
4. Benatar M, Wuu J, Lombardi V, et al. Neurofilaments in pre-symptomatic ALS and the impact of genotype. Amyotroph Lateral Scler Frontotemporal Degener 2019;20:538-548.
5. Benatar M, Cai X, McDermott MP, et al. Proposed Research Criteria for Mild Motor Impairment as a Prodromal Syndrome in Amyotrophic Lateral Sclerosis. Neurology 2025;105:e213917.
6. Benjamini Y, Hochberg Y. Controlling the False Discovery Rate: A Practical and Powerful Approach to Multiple Testing. Journal of the Royal Statistical Society Series B (Methodological) 1995;57:289-300.
7. Raudvere U, Kolberg L, Kuzmin I, et al. g:Profiler: a web server for functional enrichment analysis and conversions of gene lists (2019 update). Nucleic acids research 2019;47:W191-W198.
8. Szklarczyk D, Kirsch R, Koutrouli M, et al. The STRING database in 2023: protein-protein association networks and functional enrichment analyses for any sequenced genome of interest. Nucleic acids research 2023;51:D638-D646.
9. Wood S. Generalized Additive Models: An Introduction with R, 2nd Edition ed. New York: Chapman and Hall/CRC, 2017.
10. Marra G, Wood S. Coverage Properties of Confidence Intervals for Generalized Additive Model Components. Scandinavian Journal of Statistics 2012;39.
11. Vatcheva KP, Lee M, McCormick JB, Rahbar MH. Multicollinearity in Regression Analyses Conducted in Epidemiologic Studies. Epidemiology (Sunnyvale) 2016;6.
12. Staffaroni AM, Quintana M, Wendelberger B, et al. Temporal order of clinical and biomarker changes in familial frontotemporal dementia. Nature medicine 2022;28:2194-2206.
13. Bycroft C, Freeman C, Petkova D, et al. The UK Biobank resource with deep phenotyping and genomic data. Nature 2018;562:203-209.
14. Sudlow C, Gallacher J, Allen N, et al. UK biobank: an open access resource for identifying the causes of a wide range of complex diseases of middle and old age. PLoS Med 2015;12:e1001779.
15. Sun BB, Chiou J, Traylor M, et al. Plasma proteomic associations with genetics and health in the UK Biobank. Nature 2023;622:329-338.
16. Smith EN, Lee J, Prilutsky D, et al. Plasma neurofilament light levels show elevation two years prior to diagnosis of amyotrophic lateral sclerosis in the UK Biobank. Amyotroph Lateral Scler Frontotemporal Degener 2023:1-7.
17. Landrum MJ, Lee JM, Benson M, et al. ClinVar: improving access to variant interpretations and supporting evidence. Nucleic acids research 2018;46:D1062-D1067.
18. Amyotrophic Lateral Sclerosis Spectrum Disorders Gene Curation Expert Panel. Gene-Disease Validity Classifications [online]. Available at: https://search.clinicalgenome.org/kb/affiliate/10096?page=1&size=25&search=. Accessed November 17, 2025.
19. Gao J, Douglas AGL, Chalitsios CV, et al. Neurodegenerative disease in C9orf72 repeat expansion carriers: population risk and effect of UNC13A. Brain : a journal of neurology 2025;148:3865-3871.
