# Supplementary material for: Longitudinal plasma proteomics predict phenoconversion to clinically manifest ALS
Source: Nat Med. 2026 Jul 27;32(9):3417–29. doi: 10.1038/s41591-026-04528-x (PMC13577924; doi:10.1038/s41591-026-04528-x)
Supplement: Supplementary file 2 [file 41591_2026_4528_MOESM2_ESM.pdf]

nature portfolio

Corresponding author(s): Michael Benatar

Last updated by author(s): Jun 16, 2026

Reporting Summary

Nature Portfolio wishes to improve the reproducibility of the work that we publish. This form provides structure for consistency and transparency in reporting. For further information on Nature Portfolio policies, see our Editorial Policies and the Editorial Policy Checklist.

Statistics

For all statistical analyses, confirm that the following items are present in the figure legend, table legend, main text, or Methods section.

n/a	Confirmed	
☐	☒	The exact sample size (*n*) for each experimental group/condition, given as a discrete number and unit of measurement
☐	☒	A statement on whether measurements were taken from distinct samples or whether the same sample was measured repeatedly
☐	☒	The statistical test(s) used AND whether they are one- or two-sided<br>*Only common tests should be described solely by name; describe more complex techniques in the Methods section.*
☐	☒	A description of all covariates tested
☐	☒	A description of any assumptions or corrections, such as tests of normality and adjustment for multiple comparisons
☐	☒	A full description of the statistical parameters including central tendency (e.g. means) or other basic estimates (e.g. regression coefficient) AND variation (e.g. standard deviation) or associated estimates of uncertainty (e.g. confidence intervals)
☐	☒	For null hypothesis testing, the test statistic (e.g. *F*, *t*, *r*) with confidence intervals, effect sizes, degrees of freedom and *P* value noted<br>*Give P values as exact values whenever suitable.*
☒	☐	For Bayesian analysis, information on the choice of priors and Markov chain Monte Carlo settings
☐	☒	For hierarchical and complex designs, identification of the appropriate level for tests and full reporting of outcomes
☐	☒	Estimates of effect sizes (e.g. Cohen's *d*, Pearson's *r*), indicating how they were calculated

Our web collection on statistics for biologists contains articles on many of the points above.

Software and code

Policy information about availability of computer code

Data collection	No custom code was used to collect data for this study.
Data analysis	All analyses were performed in R 4.4.0.<br>(1) Available to editors and reviewers during manuscript review, with an embargoed access setting: https://zenodo.org/records/17852153. Reviewers may use this link to obtain anonymous access to the full analysis pipeline in order to reproduce results.<br>(2) Analysis codes will be publicly available upon publication of the manuscript: https://github.com/ranxm2/ALS_protein_longitudinal

For manuscripts utilizing custom algorithms or software that are central to the research but not yet described in published literature, software must be made available to editors and reviewers. We strongly encourage code deposition in a community repository (e.g. GitHub). See the Nature Portfolio guidelines for submitting code & software for further information.

Data

Policy information about availability of data

All manuscripts must include a data availability statement. This statement should provide the following information, where applicable:

- Accession codes, unique identifiers, or web links for publicly available datasets
- A description of any restrictions on data availability
- For clinical datasets or third party data, please ensure that the statement adheres to our policy

Access to discovery cohort Olink proteomic data (and corresponding phenotypic data) is controlled to protect study blinding and pathogenic variant carrier

confidentiality. Requests for access to this dataset should be directed to the corresponding author (mbenatar@med.miami.edu). Data requests will be reviewed based on compliance with the Informed Consent and IRB approvals under which the original samples and phenotypic data have been collected, scientific merit and feasibility, appropriateness of the investigator's qualifications, and resources to protect the data. University of Miami may then release said data via a Data Transfer Agreement. Access to Olink data from the UK Biobank can be obtained via controlled access via their web portal (www.ukbiobank.ac.uk).

Research involving human participants, their data, or biological material

Policy information about studies with human participants or human data. See also policy information about sex, gender (identity/presentation), and sexual orientation and race, ethnicity and racism.

Reporting on sex and gender	As explained in Methods, sex (based on self-report) was included as a biological variable in all relevant analyses.
Reporting on race, ethnicity, or other socially relevant groupings	Race and ethnicity were not included in the analyses.
Population characteristics	The covariate-relevant characteristics of the study population are summarized in Table 1. Specifically, in the Discovery cohort, 27 of 59 (46%) of healthy controls, 6 of 10 (60%) pre-symptomatic carriers, 13 of 33 (39%) phenoconverters, and 20 of 35 (57%) clinically manifest ALS patients were male. Average (±SD) age at baseline was 51.3 (±12.4) years for healthy controls, 49.1 (±8.9) for presymptomatic carriers, 57.2 (±10.1) for phenoconverters, and 56.8 (±9.4) for those with clinically manifest ALS. Among pre-symptomatic carriers, 4 (40%) had an SOD1 A4V mutation, 4 (40%) had an SOD1 non-A4V mutation, and 2 (20%) had a C9orf72 repeat expansion. Among phenoconverters, these frequencies were 15 (46%), 8 (24%), and 7 (21%), respectively, with another 3 (9%) carrying a pathogenic variant in another gene. Among those with clinically manifest ALS, 1 (3%) carried an SOD1 A4V mutation, 2 (6%) an SOD1 non-A4V mutation, 6 (17%) a C9orf72 repeat expansion, and no pathogenic variant was identified in 26 (74%). In the Replication cohort (UK Biobank), 47% of healthy controls, 40% of pre-symptomatic carriers, 53% of phenoconverters, and 68% of those with clinically manifest ALS were male. Baseline ages for these four groups were 56.7 (±8.2), 55.1 (±6.3), 60.8 (±6.8) and 60.6 (±6.5) years, respectively. Among the 38 pre-symptomatic carriers, 4 (11%) carried an SOD1 non-A4 mutation and 34 (89%) a C9orf72 repeat expansion. Among the 231 phenoconverters, 1 (0.4%) carried an SOD1 non-A4V mutation, 24 (11%) C9orf72 repeat expansion, 3 (1%) a pathogenic variant in another gene, and in 203 (88%) there was no identifiable genetic cause of disease. Among those with clinically manifest disease, 2 (9%) of 22 carried a C9orf72 repeat expansion, and in 20 (91%) there was no identifiable genetic cause of disease.
Recruitment	Plasma samples for this experiment came from 3 parent studies: (1) Pre-fALS (NCT00317616) is a single-center study at the University of Miami. It recruits, from across North America, individuals who are carriers of any ALS associated pathogenic variant and who, at the time of enrollment, are clinically pre-symptomatic. Since the study covers the cost of travel, recruitment is unbiased with respect to geographical location and socioeconomic status. (2) CRiALS (NCT00136500) Biomarker is a single-center study at the University of Miami, and recruits healthy controls and patients with clinically manifest ALS primarily to serve as comparison groups for the pre-symptomatic carriers in Pre-fALS. These are mostly local recruits from South Florida. (3) PGB1 (Phenotype-Genotype-Biomarker, NCT02327845) study of the CReATe Consortium is multi-center, but those included in this experiment were all from the University of Miami site. As with all natural history studies, CRiALS Biomarker and PGB1 may be enriched for patients with slower progressing disease. Since the temporal course of biomarkers may vary based on rate of disease progression, this is a potential source of bias. However, this study focuses on the pre-symptomatic stage of disease, with biomarker trajectories minimally impacted by inclusion of data from patients with clinically manifest disease.
Ethics oversight	All 3 parent studies were approved by the University of Miami institutional review board (IRB), and all study participants provided written informed consent. The University of Miami IRB operates under the umbrella of the University of Miami Human Subjects Research Office (FWA0000224). The costs of travel (including flights, hotel, meals, and incidentals) for Pre-fALS in-person visits were covered (i.e. reimbursed) by the study, as were incidental costs (e.g. parking) for PGB1 study visits. No compensation for participation was provided.

Note that full information on the approval of the study protocol must also be provided in the manuscript.

Field-specific reporting

Please select the one below that is the best fit for your research. If you are not sure, read the appropriate sections before making your selection.

☒ Life sciences ☐ Behavioural & social sciences ☐ Ecological, evolutionary & environmental sciences

For a reference copy of the document with all sections, see nature.com/documents/nr-reporting-summary-flat.pdf

Life sciences study design

All studies must disclose on these points even when the disclosure is negative.

Sample size	An Olink experiment of 516 longitudinal plasma samples was planned, given available funds at the time. This study focused on phenoconverters; all N=33 Pre-fALS phenoconverters with at least 2 plasma collections (total 202 samples) were included. N=59 healthy controls with at least 3 plasma collections (total 126 samples) were included; if a control had more than 3 collections, the first and last collection along with a collection near the mid-point of study follow-up were included. N=35 ALS patients with at least 3 plasma collections (total 126 samples), and who were broadly representative of patients at different stages of disease, were included. N=10 pre-symptomatic participants thought to be of high risk for phenoconversion but not yet developed clinically manifest disease (total 62 samples) were also

	included; since this study focuses on the prediction of phenoconversion, any bias that this selection might have introduced, would have been towards the null. These 4 participant groups were age- and sex-matched as much as possible. See Methods for further details.
Data exclusions	None were excluded from analyses
Replication	For replication, we used UK Biobank data, the best available dataset (to our knowledge) for this purpose. That the results from our discovery cohort were only partially replicated in the UK Biobank replication cohort was likely due in part to the limitations of relying on cross-sectional proteomic data and very imprecise proxies of the timing of phenoconversion in the latter, as well as differences in proteomic platforms between the two cohorts.
Randomization	There was no intervention or experimental group involved, and thus no randomization at the participant level. However, in the Olink experiment, the 516 plasma samples were run on 6 plates of 86 samples each. Longitudinal samples from the same participant were included on the same plate. In addition, plate assignment was devised to achieve a balanced design, with similar distributions of participant group (control, pre-symptomatic, phenoconverter, clinically manifest), age, and sex on each of the 6 plates.
Blinding	Proteomic data were generated blind to participant group.

Reporting for specific materials, systems and methods

We require information from authors about some types of materials, experimental systems and methods used in many studies. Here, indicate whether each material, system or method listed is relevant to your study. If you are not sure if a list item applies to your research, read the appropriate section before selecting a response.

Materials & experimental systems

n/a	Involved in the study
☒	☐ Antibodies
☒	☐ Eukaryotic cell lines
☒	☐ Palaeontology and archaeology
☒	☐ Animals and other organisms
☐	☒ Clinical data
☒	☐ Dual use research of concern
☒	☐ Plants

Methods

n/a	Involved in the study
☒	☐ ChIP-seq
☒	☐ Flow cytometry
☒	☐ MRI-based neuroimaging

Clinical data

Policy information about clinical studies
All manuscripts should comply with the ICMJE guidelines for publication of clinical research and a completed CONSORT checklist must be included with all submissions.

Clinical trial registration	This is a natural history study, not an interventional trial. Nonetheless, the 3 parent studies are registered on clinicaltrials.gov: Pre-fALS (NCT00317616), CRiALS (NCT00136500), CReATe PGB1 (NCT02327845).
Study protocol	This is not a clinical trial, and this study does not have a protocol, as the biological samples and accompanying data derived from 3 parent natural history studies.
Data collection	Location: At the study center (Feb 2011 and before: Emory University; thereafter, due to PI move: University of Miami), in dedicated research space. Period of recruitment and data collection: Dec 2007 - Jan 2025.
Outcomes	In this analysis of proteomic data from a natural history study, phenoconversion (i.e., the development of clinically manifest disease) is the outcome measure of interest.

Plants

Seed stocks	
Novel plant genotypes	
Authentication	
